# Efficacy and safety of inclisiran in stroke or cerebrovascular disease prevention: a systematic review and meta-analysis of randomized controlled trials

**DOI:** 10.3389/fphar.2023.1158274

**Published:** 2023-06-13

**Authors:** Min Luo, Yihan Liu, Xinyi Xu, Kai Liu, Chao Shen, Haoyang Hu, Zhiyao He, Fengbo Wu

**Affiliations:** ^1^ Department of Pharmacy, West China Hospital, Sichuan University, Chengdu, China; ^2^ West China School of Pharmacy, Sichuan University, Chengdu, Sichuan, China; ^3^ Department of Cardiology, West China Hospital, Sichuan University, Chengdu, China

**Keywords:** inclisiran, stroke, ASCVD, systematic review, meta-analysis, rct

## Abstract

**Aims:** As the impact of inclisiran in stroke prevention in atherosclerotic cardiovascular disease (ASCVD) patients or those at high risk of ASCVD is still unclear, we conducted a systematic review and meta-analysis of randomized controlled trials (RCT) to quantify the effectiveness of inclisiran in stroke prevention in these patients.

**Methods:** Literature research was conducted in four electronic databases (PubMed, EMBASE, Web of Science, CENTRAL) and two clinical trials registers (ClinicalTrials.gov, WHO ICTRP) from the inception of the study to 17 October 2022, and was updated by the end of the study on 5 January 2023. Two authors independently screened the studies, extracted the data, and assessed the bias. The risk of bias was assessed using the Cochrane risk-of-bias tool for randomized trials (RoB 2). The intervention effect was estimated by calculating risk ratio (RR), weighted mean difference (WMD), and 95% confidence interval (CI) with R 4.0.5. Sensitivity analysis by changing meta-analysis model was also performed to test the robustness of the pooled results. If this was not possible, a descriptive analysis was conducted.

**Results:** Four RCTs (*n* = 3,713 patients) were rated as high-risk bias. Meta-analysis of three RCTs (ORION-9, ORION-10, and ORION-11) showed that inclisiran reduced myocardial infarction (MI) risk by 32% (RR = 0.68, 95%CI = 0.48–0.96) but did not reduce stroke (RR = 0.92, 95%CI = 0.54–1.58) and major cardiovascular events (MACE) (RR = 0.81, 95%CI = 0.65–1.02) risk. Sensitivity analysis results were stable. Safety was similar to the placebo group but had frequent injection-site reactions (RR = 6.56, 95%CI = 3.83–11.25), which were predominantly mild or moderate. A descriptive analysis of one RCT (ORION-5) was conducted due to different study designs, and suggested that inclisiran might be given semiannually from the beginning.

**Conclusion:** Inclisiran is not beneficial for stroke or MACE prevention in ASCVD or patients at high risk of ASCVD but is associated with the reduction of MI. Given the limited number and quality of the available studies and the lack of a standardized definition for cardiovascular events, further studies are essential for confirming the results.

## 1 Introduction

Stroke is a neurological disease in which brain tissue is damaged due to the sudden rupture of a blood vessel or a blood vessel embolism, which can lead to sudden death (Kuriakose and Xiao, 2020) and can also generate depression (Medeiros et al., 2020) and dementia (Pasi et al., 2012). Stroke is characterized by high incidence, high disability, high recurrence, high death, and high burden. According to the statistics, in 2021, stroke remained the second-leading cause of death and the third-leading cause of death and disability combined in the world. Atherosclerotic cardiovascular disease (ASCVD) refers to the accumulation of plaque in the artery, with a risk of bleeding within the plaque, necrotic core rich in lipids, and fibrous cap rupture (Deng et al., 2020), which can lead to the occurrence of acute coronary syndrome, angina pectoris, stroke, transient ischemic attack (TIA), and peripheral artery disease (Rogers and Baker, 2020). The risk of stroke is further increased when patients have GBD 2019 Stroke Collaborators. (2021), so it is particularly important to prevent the occurrence of stroke in ASCVD and in patients at high risk of ASCVD. Lipid, especially low-density lipoprotein cholesterol (LDL-C), is the most prominent risk factor for ASCVD (Khatana et al., 2020). Lipid detection in stroke patients showed that the levels of total cholesterol (TC), triglyceride (TG), LDL-C, apolipoprotein A (Apo A), apolipoprotein B (Apo B), apolipoprotein E (Apo E), and lipoprotein a (Lp [a]) were significantly higher, while high-density lipoprotein cholesterol (HDL-C) was significantly lower (Yuan et al., 2015). Moreover, low HDL-C (<0.90 mmol/L) and high TG (>2.30 mmol/L) were associated with a two-fold increased risk of death in stroke (Kuriakose and Xiao, 2020). The LDL-C level was positively correlated with the occurrence of ischemic stroke (Holmes et al., 2018) and associated with long-term post-stroke mortality (Xing et al., 2016). Therefore, lipid-regulating therapy may play a key role in stroke prevention, especially for patients with ASCVD or at high risk of ASCVD.

Currently, statins are recommended as the first choice to reduce LDL-C in patients with increased risk of stroke in stroke prevention guidelines (Amarenco et al., 2004), and the benefits of more intensive LDL-C-lowering statin-based therapies for recurrent stroke risk reduction might be more favorable than the less intensive LDL-C-lowering statin-based therapies (Lee et al., 2022). The preventive effect of non-statin drugs, such as ezetimibe and proprotein convertase subtilisin/kexin type 9 (PCSK9) inhibitors, on stroke also produced significant benefits in studies (Hackam and Hegele, 2022), and compared to low-risk populations, the effect was only seen in high-risk ASCVD populations who had received a maximum tolerated dose of statins or who were intolerant to statins (Khan et al., 2022). Statins combined with ezetimibe or PCSK9 monoclonal antibody could reduce the risk of stroke by 26% (Khan et al., 2022). A 2021 guideline for the prevention of stroke in patients with stroke and TIA (Kleindorfer et al., 2021) recommended that for patients with a very high risk of stroke who have been treated with the combination of a maximum tolerated dose of statins and ezetimibe but whose LDL-C level is still not up to the standard, PCSK9 monoclonal antibody is a feasible therapy to prevent cardiovascular events (CVEs). However, for some patients, even after treatment with the previously mentioned drugs, the lipid level still fails to reach the standard. Furthermore, as a disease requiring long-term drugs for prevention, the incidence of stroke is higher in low and middle-income countries, and one-third of patients discontinue the use of one or more prevention drugs approximately 1 year after stroke (2021). Therefore, the development of a new mechanism for lipid-regulating treatments with better economic effectiveness and compliance is of great significance.

The degradation of LDL-C requires the action of LDL receptor (LDL-R) in the liver, and PCSK9 can compete with LDL-C, bind, and cause the LDL-R to be degraded by lysosomes (Moustafa and Testai, 2021). This process reduces the density of LDL-R on the cell surface and increases the LDL-C level. Therefore, inhibiting the synthesis of PCSK9 is an important mechanism in the development of lipid-regulating drugs. PCSK9 monoclonal antibodies were designed to reduce LDL-C by preventing the combination of and interaction between PCSK9 and LDL-R (Go and Mani, 2012). Over the past few decades, the birth of ribonucleic acid (RNA) interference (RNAi)-based therapeutics has ushered in a new era of drug development (Gangopadhyay and Gore, 2022). Inclisiran, the first small interfering RNA (siRNA) drug in the cardiovascular field and a new PCSK9 inhibitor, is an example of nucleic acid therapeutics.

Inclisiran consists of a passenger strand and a guide strand, with a triantennary N-acetylgalactosamine (tri-GalNAc) conjugated to the end. As an established liver targeting technique, the tri-GalNAc can specifically bind to the asialoglycoprotein receptor, which is only highly expressed in the liver (Springer and Dowdy, 2018). In this way, after inclisiran is specifically introduced into liver cells, with the assistance of the passenger strand, the guide strand identifies the information of PCSK9 message RNA (mRNA) and forms RNA-induced silencing complexes (RISC) with some enzymes inside the cell. RISC performs the cleavage and degradation of PCSK9 mRNA to block the synthesis of PCSK9 and reduce the LDL-C level (Fitzgerald et al., 2017; Khvorova, 2017).

Inclisiran has now been proven to have effective and long-lasting effects, with a single subcutaneous injection reducing the LDL-C level for 6 months. Compared with PCSK9 monoclonal antibodies, inclisiran is closer to the source of dyslipidemia, and the administration schedule (twice a year) also allows healthcare providers to manage ASCVD patients during their regular visits and improve compliance (Soffer et al., 2022).

Inclisiran has been shown to have a strong and consistent lipid-lowering effect in some randomized controlled trials (RCT). RNAi therapy may be used if statins are not effective in reducing lipid levels or are intolerant. Therefore, inclisiran may be of great significance in stroke prevention. However, the efficacy and safety of inclisiran in stroke prevention in ASCVD or ASCVD high-risk patients remain unclear. Therefore, we conducted a systematic review and meta-analysis of the available evidence from RCTs to quantify the effectiveness of inclisiran in the prevention of the risk of stroke in patients with ASCVD or at high risk of ASCVD.

## 2 Methods

This study was reported according to the Preferred Reporting Items for Systematic Reviews and Meta-Analyses (PRISMA) 2020 statement (Page et al., 2021) (the PRISMA 2020 checklist is shown in Supplementary Table S1) (Shamseer et al., 2015). We have registered this study in the International Prospective Register of Systematic Reviews (PROSPERO) (registration number: CRD42022374280).

### 2.1 Literature search and inclusion criteria

The databases Pubmed, Embase, the Cochrane Central Register of Controlled Trials (CENTRAL), and Web of Science were researched from the study’s inception to 17 October 2022 for potentially relevant studies, without language restrictions, using the search terms: exposure (Cardiovascular Diseases or Heart Disease Risk Factors or stroke or Cerebrovascular Disorders or Ischemic Attack), intervention (Inclisiran or ALN-60212 or ALN-PCS or ALN-PCSsc), and study (randomized controlled trial or controlled clinical trial or randomized). We also searched two clinical trials registers, ClinicalTrials.gov (https://ClinicalTrials.gov/) and WHO ICTRP (https://trialsearch.who.int/), for RCTs using the search terms: intervention (ALN-PCSsc or ALN-60212 or PCSK9si KJX-839 or inclisiran or small interfering RNA or RNAi or siRNA or RNA, Small Interfering) and filters (with results). By the end of the study (5 January 2023) and the revision of the study (6 March 2023), we retrieved and updated the inclusions. The complete search terms and records are provided in Supplementary Table S2. Additionally, we manually examined the reference lists of retrieved studies to identify additional relevant literature.

The studies were included if they met the following criteria: (1) the enrolled patients suffered from ASCVD or were at high risk of ASCVD (Supplementary Table S3); (2) the intervention was inclisiran used alone or in combination with other lipid-regulating drugs. The duration of inclisiran treatment met the current standard administration: 300 mg dosage of inclisiran sodium or 284 mg dosage of inclisiran administered as a single subcutaneous injection initially, then again at 3 months, and then every 6 months, all subjects receiving at least three doses; (3) the control group was given a placebo or other drugs; (4) the outcomes include at least one of the following: stroke, cerebrovascular disease, major adverse cardiovascular events (MACE), all-cause mortality, change in serum LDL-C, PCSK9, and other lipid parameters (TG, TC, HDL-C, et al.) from baseline to the last available follow-up, adverse events (AE), treatment-emergent adverse events (TEAE), TEAE leading to discontinuation of treatment, and serious adverse events (SAE) (the definition of SAE is shown in Supplementary Table S4 and is consistent with the Good Clinical Practice Guideline of International Conference on Harmonization); (5) the study was designed as an RCT. Studies were excluded if they met one of the following criteria: (1) the results were not yet available or the full text could not be accessed; (2) the articles were conference articles, letters, qualitative studies, reviews, commentaries, pilot studies, or protocols.

All titles and abstracts of the studies were downloaded and imported into Endnote X9. Study selection was independently conducted by two review authors (ML and YL) after deleting the duplications by automatic tool and by humans. The irrelevant studies were excluded by screening the titles and abstracts first and then reviewing the full text of each literature to select the included studies in conformity with the eligibility criteria. If there were discrepancies in any details of the literature, the third reviewer (XX) made the necessary decisions after discussion.

### 2.2 Data extraction and outcome assessments

Two authors (ML and YL) independently extracted data from eligible studies using a data extraction form set in advance. The contents of the data extraction form included author, published year, name of RCT, data source (from literature or clinical trials registers), ClinicalTrials.gov Identifier, study design, duration of follow-up, participants (sample size, diagnosis, background therapy), intervention group (age, sex, sample size, baseline LDL-C mg/dL), control group (age, sex, sample size, baseline LDL-C mg/dL), outcomes (primary endpoints, key secondary endpoints, prespecified exploratory endpoints, safety).

The primary outcomes of our study were the occurrence of stroke or cerebrovascular disease and MACE. The secondary outcomes were all-cause mortality, change in serum LDL-C and PCSK9 levels from baseline to the last available follow-up, change from baseline in other lipid parameters, and TEAEs, TEAE leading to discontinuation of treatment, and SAEs.

The primary outcomes were defined using the standardized Medical Dictionary for Regulatory Activities (MedDRA) queries (SMQs) from MedDRA version v20.1. Stroke and cerebrovascular disease were defined as central nervous system vascular conditions (SMQ), which can also be subdivided into “ischaemic central nervous system vascular conditions (SMQ),” “hemorrhagic central nervous system vascular conditions (SMQ),” and “central nervous system vascular disorders, not specified as hemorrhagic or ischaemic (SMQ).” MACE was defined as the composite of “cardiovascular cause death,” “myocardial infarction (MI),” “stroke,” “cardiac arrest,” and “cardiac failure.” The included SMQ and preferred term (PT) for each outcome can be found in Supplementary Table S4.

### 2.3 Risk of bias

The risk of bias in each included study was also independently assessed by two authors (ML and YL) using the Cochrane risk-of-bias tool for randomized trials (RoB 2) recommended in the Cochrane, 2022. The discrepancies were resolved by the third author (XX). RoB 2 is structured into a fixed set of domains of bias, focusing on different aspects of trial design, conduct, and reporting. It includes five domains: “bias arising from the randomization process,” “bias due to deviations from intended interventions,” “bias due to missing outcome data,” “bias in measurement of the outcome,” and “bias in selection of the reported result.” Each domain has a series of signaling questions that need to be judged and responded to objectively by the authors based on the actual content of the studies. There are five response options in each domain: “Yes (Y),” “Probably yes (PY),” “Probably no (PN),” “No (N),” and “No information (NI).” Once the signaling questions are answered, a risk-of-bias judgment can be reached and one of three levels can be assigned to each domain: “Low risk of bias,” “Some concerns,” or “High risk of bias.”

### 2.4 Data synthesis and analysis

The heterogeneity tests and meta-analysis were conducted with the “meta-package” of R statistical language version 4.0.5. Heterogeneity between studies was assessed using the I^2^ statistic and Cochran’s Q test, with I^2^ >50% and *p*-value < 0.10 considered as having high heterogeneity. If high heterogeneity was present between studies, we used a random-effects model or provided a narrative overview. If heterogeneity was not identified, we computed pooled estimates of the treatment effect for each outcome under a fixed-effect model. For dichotomous outcome measures (such as cardiovascular outcomes), we calculated a pooled estimate of the treatment effect for each outcome across trials using the risk ratio (RR) and 95% confidence intervals (CI) according to the Mantel-Haenszel method. For continuous outcomes (such as LDL-C and PCSK9 levels), we used the weighted mean difference (WMD) with 95%CI. The overlap of intervention effects was shown using a forest plot, and differences with *p* values of <0.05 were considered statistically significant. In addition, we conducted a sensitivity analysis by changing pooled model to test the robustness and reliability of the pooled results. If the number of included studies was ≥10, a funnel plot or an Egger’s test was used to assess publication bias (2016; Riley et al., 2019), otherwise, it was regarded as the existence of publication bias.

## 3 Results

### 3.1 Study selection

**FIGURE 1:**
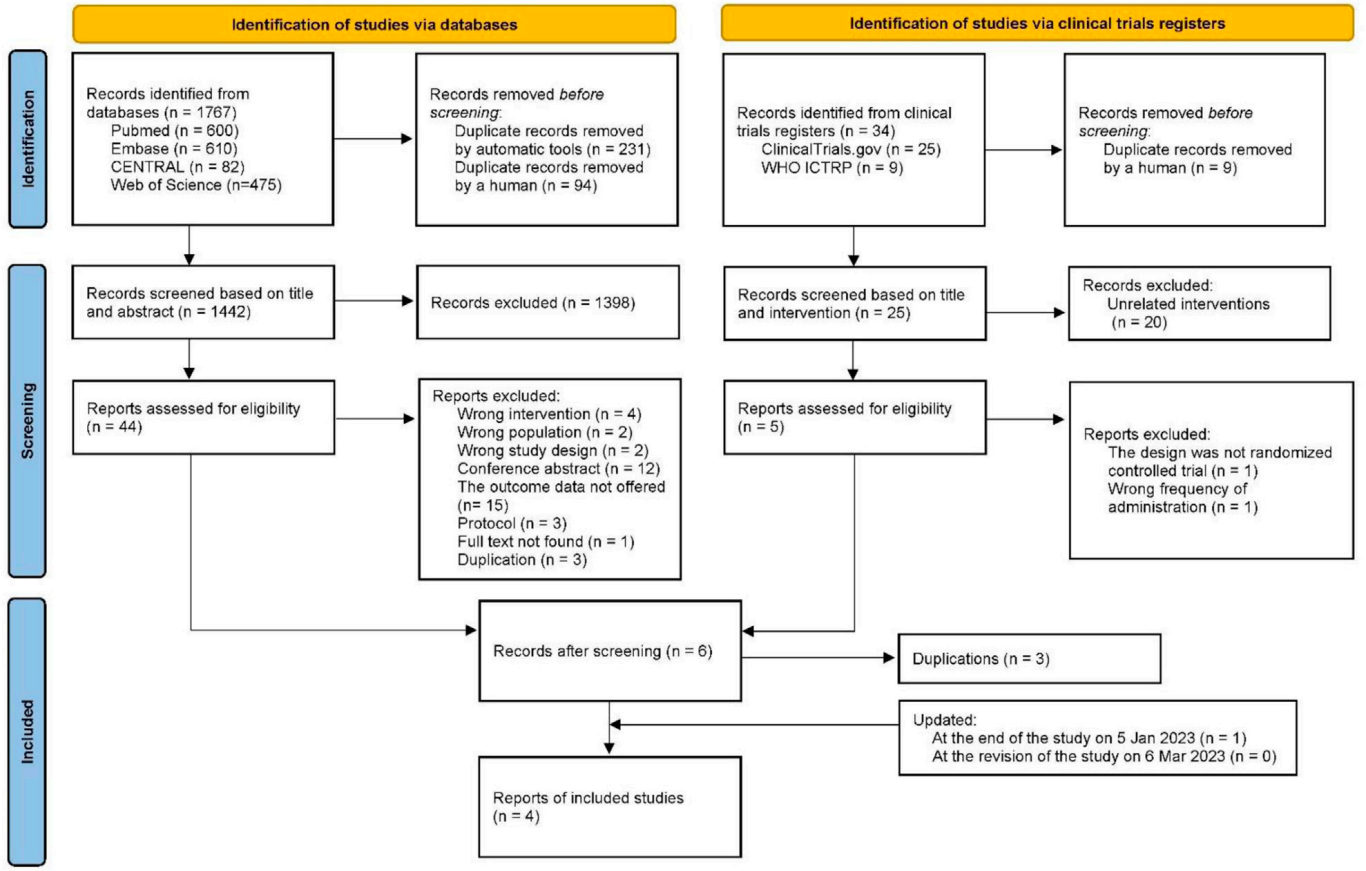
PRISMA flowchart.

In the initial search, 1,767 and 34 records were retrieved from four electronic databases and two clinical trial registers, respectively. After removing 334 records using the automatic tool and manual de-duplication, 1,418 records were excluded according to the review of the titles, abstracts, and interventions. Subsequently, 44 records from databases and 5 records from registers underwent full-text review. Finally, three studies, ORION-9 (Raal et al., 2020), ORION-10 (Ray et al., 2020), and ORION-11 (Ray et al., 2020), were eligible for data extraction and quantitative analysis. The search and selection processes are shown in Figure 1. At the end of the study on 5 January 2023, we searched the clinical registers again and found that a new RCT meeting the criteria, ORION-5, was included in our review. Additionally, we searched all databases again when we revised this study (6 March 2023), and no new RCTs were found that met the criteria.

### 3.2 Characteristics of included studies

The characteristics of the studies are reported in Table 1. Except for part 2 in ORION-5, the studies were double-blind, randomized, placebo-controlled international multicenter clinical trials conducted in many different countries or sites, and the outcomes were published between 2020 and 2022. In total, data from 3,713 patients were included. All included subjects were aged >18 years and were mainly middle-aged and elderly. The subjects were predominantly White (>80%). Patients in four studies had histories of disease involving diagnoses of heterozygous familial hypercholesterolemia (HeFH), ASCVD, ASCVD or an ASCVD risk equivalent, and homozygous familial hypercholesterolemia (HoFH), respectively, and their history of treatment involved the maximally tolerated statin with or without other lipid-lowering therapy. Interventions in ORION9 (Raal et al., 2020), ORION-10 (Ray et al., 2020), and ORION-11 (Ray et al., 2020) were inclisiran 300 mg on day 1 and day 90, then every 6 months, with an 18-month follow-up period. ORION-5 consisted of two parts. Part 1 was a 6-month double-blind period in which subjects were randomized to receive either inclisiran 300 mg or a placebo on day 1 and day 90. Part 2 was an 18-month open-label follow-up period, and all subjects from part 1, including the experimental group and control group received inclisiran 300 mg on day 180 and then every 6 months. Outcomes mainly included efficacy and safety outcomes. The efficacy outcomes mainly included the changes in LDL-C and other lipid levels and PCSK9 levels. ORION-9 (Raal et al., 2020), ORION-10 (Ray et al., 2020), and ORION-11 (Ray et al., 2020) also included exploratory cardiovascular outcomes. According to the different interventions, a meta-analysis was conducted in ORION-9, ORION-10, and ORION-11, and a descriptive analysis was conducted in ORION-5.

**TABLE 1:** The characteristics of included studies^a^.

Author/Published year/Name	ClinicalTrials.gov Identifier (NCT number)	Study design	Locations	Duration of follow-up (months)	Participants				Intervention group				
					Sample size (N)	Diagnosis	Background therapy	White people (N)	Age, mean (SD) (years)	Male/Female	Sample size (N)	Treatment	Baseline LDL-C, mean (SD), mg/dL
Raal et al. (2020) ORION-9	NCT03397121	RCT (DB, PC)	United States, Canada, Europe, South Africa	18	482	HeFH	Maximally tolerated statin with/without other LLT	453	54.4 (12.48)	112/130	242	Inclisiran 300 mg at day 1, day 90, then every 6 months	151.4 (50.4)
Ray et al. (2020) ORION-10	NCT03399370	RCT (DB, PC)	United States	18	1561	ASCVD		1311	66.4 (8.9)	535/246	781	Inclisiran 300 mg at day 1, day 90, then every 6 months	104.5 (39.6)
Ray et al. (2020) ORION-11	NCT03400800	RCT (DB, PC)	Europe, South Africa	18	1617	ASCVD or an ASCVD risk equivalent		1587	64.8 (8.3)	579/231	810	Inclisiran 300 mg at day 1, day 90, then every 6 months	107.2 (41.8)
2022 ORION-5	NCT03851705	Part1: RCT (DB, PC)	Hong Kong, Israel, Russian Federation, Serbia, South Africa, Taiwan, Turkey, Ukraine	Part 1: 6 Part 2: 18	Part 1: 53 Part 2: 47	FoFH		48	Unclear	Total: 14/23	Part 1: 34 Part 2: 29	Part 1: Inclisiran 300 mg at day 1, day 90	Unclear
		Part 2: OL										Part 2: Inclisiran 300 mg at day 270, day 450, and day 630	

Author/Published year/Name	Control group					Outcomes			
	Age, mean (SD) (years)	Male/Female	Sample size (N)	Treatment	Baseline LDL-C, mean (SD), mg/dL	Primary endpoints	Key secondary endpoints	Prespecified exploratory endpoints	Safety
Raal et al. (2020) ORION-9	55.0 (11.81)	115/125	240	0.9% NaCl on day 1, day 90, then every 6 months	154.7 (58.0)	Percentage change in LDL-C from baseline to day 510 and the time-adjusted percentage change in LDL-C from baseline between day 90 and day 540	Absolute change from baseline to day 510 and time-adjusted absolute change from baseline between day 90 and day 540 in LDL-C; Percentage change in PCSK9, TC, Apo B, Non-HDL-C from baseline to day 510	Proportion of patients who met the lipid targets for their level of cardiovascular risk and the treatment response according to the underlying genotype of FH	Frequent AEs, Serious AEs, Other cardiovascular AEs (Prespecified exploratory cardiovascular event, Fatal or nonfatal MI, Fatal or nonfatal stroke), Protocol-defined injection-site reaction, Laboratory results et al
Ray et al. (2020) ORION-10	65.7 (8.9)	548/232	780	0.9% NaCl on day 1, day 90, then every 6 months	104.8 (37.0)			MedDRA defined cardiovascular basket of non-adjudicated terms, including those classified within cardiac death, and any signs or symptoms of cardiac arrest, nonfatal MI, or stroke	
Ray et al. (2020) ORION-11	64.8 (8.7)	581/226	807	0.9% NaCl on day 1, day 90, then every 6 months	103.7 (36.4)				
2022 ORION-5	Unclear	Total: 8/11	Part 1: 19 Part 2: 18	Part 1: 0.9% NaCl on day 1, day 90;	Unclear	Percentage change in LDL-C from baseline to day 150	Percentage change and absolute change in LDL-C, PCSK9, TC, Apo B, Non-HDL-C, HDL-C, VLDL-C, Apo-A1, Lp(a), hsCRP from baseline to day 90, 150, 180, 330, 450, 510, 630, 690, and 720; Individual responsiveness of subjects (Number of subjects reaching on treatment LDL-C of <25 mg/dL, <50 mg/dL, <70 mg/dL, and <100 mg/dL up to Day 180 and 720); Proportional responsiveness (Number of participants in each group who attain global lipid targets for their indication) et al	Unclear	All-Cause Mortality, Serious AEs, Other (Not include serious) AEs
				Part 2: Inclisiran 300 mg on day 270, day 450, and day 630					

^a^In Table 1, RCT, randomized controlled trial; DB, double-blind; PC, placebo-controlled; OL, open-label; N, number; FH, familial hypercholesterolemia; HeFH, heterozygous FH; ASCVD, atherosclerotic cardiovascular disease; FoFH, homozygous FH; LLT, lipid-lowering therapy; SD, standard deviation; LDL-C, low-density lipoprotein cholesterol; PCSK9, proprotein convertase subtilisin/kexin type 9; TC, total cholesterol; Apo B, Apolipoprotein B; HDL-C, high-density lipoprotein cholesterol; Non-HDL-C, Non-HDL cholesterol; VLDL-C, Very-Low-Density Lipoprotein Cholesterol; Apo-A1, Apolipoprotein A-1; Lp(a) Lipoprotein(a); hsCRP, High-Sensitivity C-Reactive Protein; AEs, adverse events; MI, myocardial infarction.

### 3.3 Risk of bias

**FIGURE 2:**
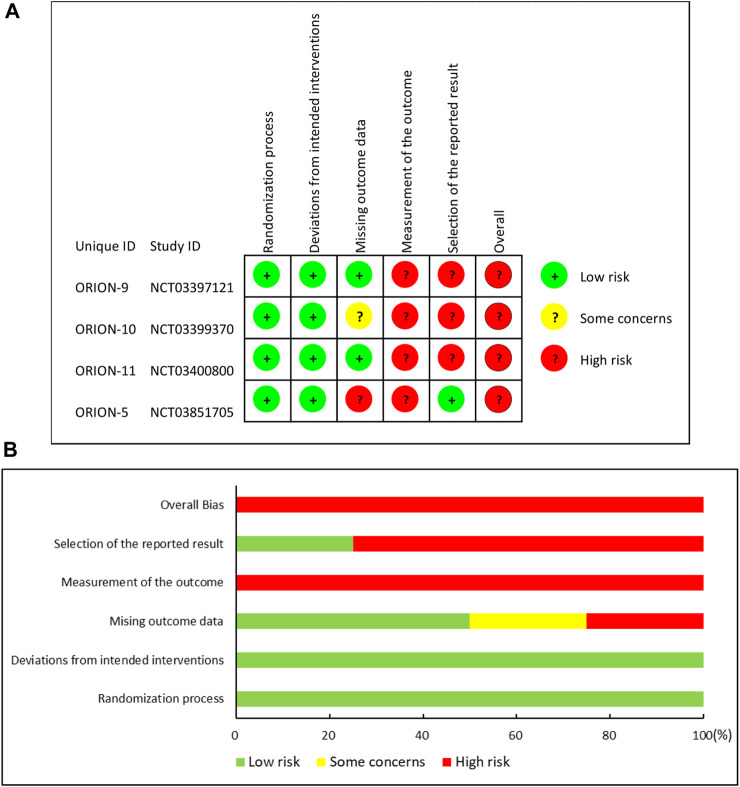
Risk of bias summary: **(A)** “traffic light” plots of the domains; **(B)** Weighted bar plots of the distribution of risk.

According to RoB 2, the included RCTs all showed a high overall risk of bias. The assessment in each domain and the summary of the risk of bias are presented in Figure 2.

More specifically, three RCTs [ORION-9 (Raal et al., 2020), ORION-10 (Ray et al., 2020), and ORION-11 (Ray et al., 2020)] and part 1 of ORION-5 were all double-blind, randomized, placebo-controlled trials. Researchers clearly described their randomization method and allocation concealment: randomization was conducted via an automated interactive response technology to assign subjects to investigational products. Study medication was blinded before distribution to the site. Each investigational product vial contained a yellow shroud to blind it. Four studies (Raal et al., 2020; Ray et al., 2020) did not mark out the differences between the patients at baseline, and intent-to-treat (ITT) analysis was used to conduct the analysis. Although part 2 in ORION-5 was an open-label follow-up period, the subjects were the continuation in part 1, and the treatment of the intervention group and control group were the same, so we considered that even if part 2 did not use blinding, the bias in the outcomes would be negligible. Therefore, they were rated as having a low risk of bias in the randomization process and deviations from intended interventions. In the assessment of bias due to missing outcome data, two studies (Raal et al., 2020; Ray et al., 2020) were rated as low risk because the data missing rate was less than 5% and the number of dichotomous outcome events was significantly greater than the missing data. The remaining two studies (Ray et al., 2020) were rated as being of some concern or being at high risk of bias because they did not meet the above conditions and the reason for missing data in the intervention and control groups did not match. In addition, four studies (Raal et al., 2020; Ray et al., 2020) were rated as having a high risk of outcome measurement bias because they did not describe the blinding to outcome assessors, and as they were all international multicenter clinical trial studies, the subjective judgment of different outcome assessors might lead to bias. Finally, three studies (Raal et al., 2020; Ray et al., 2020) were rated as having a high risk of bias in the selection of the reported result because the supplementary appendix indicated that several analysis techniques were utilized to assess the efficacy of inclisiran. Treatments were compared utilizing two-sample t-tests, analysis of covariance models (ANCOVA), and mixed models for repeated measures. However, only ANCOVA results were reported. One study was rated as having a low risk due to the match of outcomes reported and the statistical methods they published.

Generally, the overall risk of bias in included studies was assessed as a high risk of bias.

### 3.4 Meta-analysis results

#### 3.4.1 Primary outcomes

We extracted data on stroke and MACE from safety reports. Since the intervention method of ORION-5 was different from others, we only conducted a meta-analysis on the data of ORION-9 (Raal et al., 2020), ORION-10 (Ray et al., 2020), and ORION-11 (Ray et al., 2020), and a descriptive analysis was conducted for ORION-5, 2023. The safety population in three studies included a total of 3,655 patients [inclisiran (*n* = 1,833); placebo (*n* = 1,822)].

**FIGURE 3:**
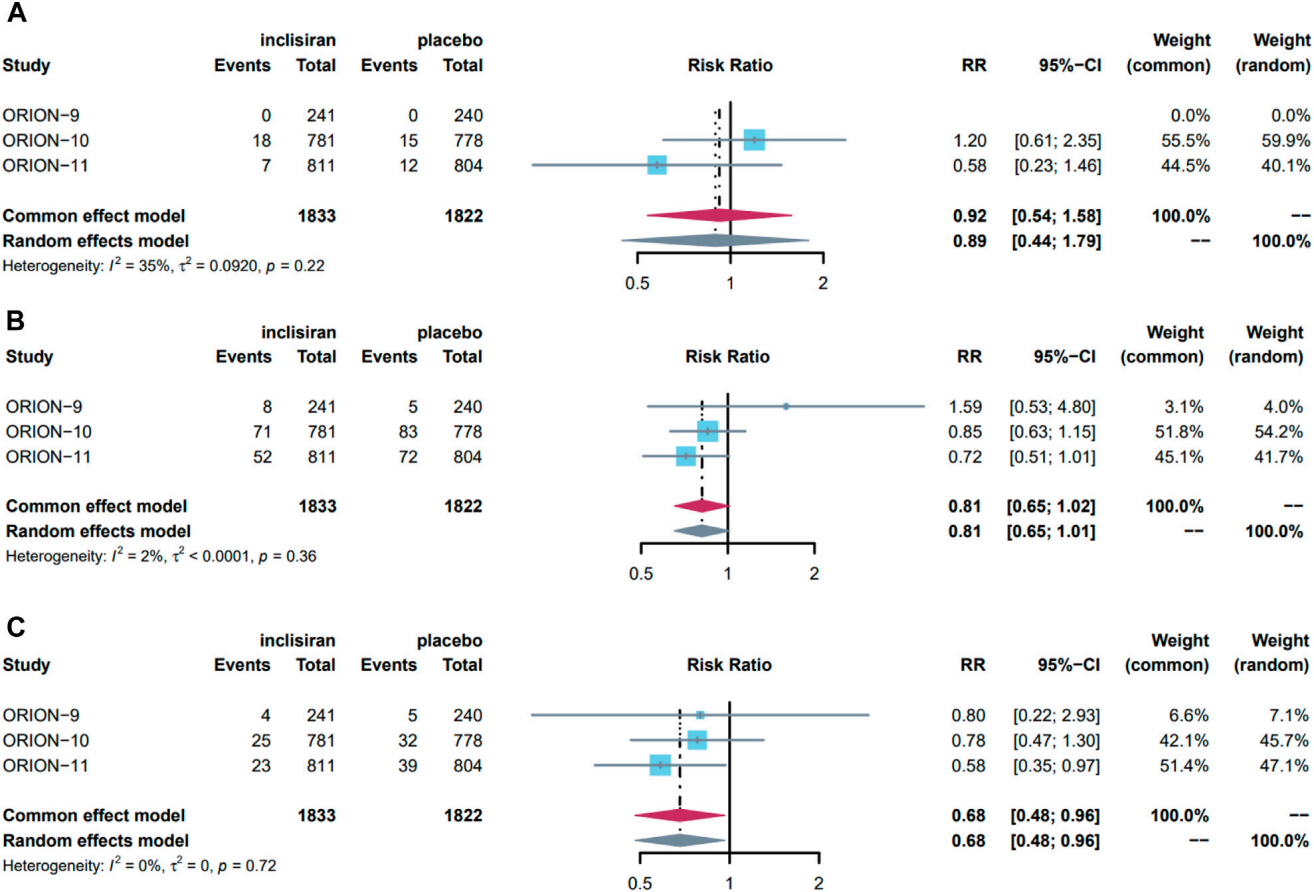
Forest plot of the effect of inclisiran in stroke, MACE, and MI, pooled using common-effects meta-analysis: **(A)** effect on stroke; **(B)** effect on MACE; **(C)** effect on MI. Overall, three studies were included in this meta-analysis. The maroon diamond represents the pooled difference using a random effects model for each subgroup and for the total. Heterogeneity of outcomes is represented by I^2^ values (%) with *p* values reported for the χ^2^ test for heterogeneity. The gray diamond represents the result of changing pooled model used for sensitive analysis. MACE major cardiovascular events, MI myocardial infarction, RR risk ratio, CI confidence interval.

Events of stroke were reported in all three studies. Stroke occurred in 25 (1.3%) patients in the experimental group and 37 (1.5%) patients in the control group. Due to a low level of heterogeneity (I^2^ = 35% < 50%, *p* = 0.22 > 0.10), we used the common-effect model to pool and analyze the data. The results showed that inclisiran did not reduce the risk of stroke (RR = 0.92, 95%CI = 0.54–1.58, *p* = 0.76) (Figure 3A). The pooled RR of ischaemic stroke and hemorrhagic stroke were 1.33 (95%CI = 0.72–2.34, *p* = 0.36) and 0.62 (95%CI = 0.23–1.63, *p* = 0.33), respectively. Sensitivity analysis showed that using the random-effect model did not reverse the pooled results, indicating that the results were stable (Supplementary Figure S1).

We conducted a meta-analysis of MACE and its subdivided components. MACE occurred in a total of 131 (7.10%) patients in the experimental group and 160 (8.8%) patients in the control group. As there was a low level of heterogeneity (I^2^ = 2% < 50%, *p* = 0.36 > 0.10), the data were pooled using a common-effect model and showed that inclisiran intervention did not significantly reduce the risk of MACE (RR = 0.81, 95%CI = 0.65–1.02, *p* = 0.07) (Figure 3B). Sensitivity analysis showed stability due to the consistent result after changing to the random-effect model.

In the component events of MACE, 17 (0.93%) and 15 (0.82%) patients in the experimental group and control group had CVE death, 52 (2.80%) and 76 (4.20%) patients had MI, 25 (1.30%) and 37 (1.50%) patients had stroke, 6 (0.33%) and 1 (0.05%) patient had cardiac arrest, and 31 (1.70%) and 41 (2.30%) patients had heart failure, respectively. In these events, the heterogeneity of the studies was low, so the common-effect model was used for analysis. Overall, inclisiran was a protective factor for MI, reducing the risk of MI by 32% (RR = 0.68, 95%CI = 0.48–0.96, *p* = 0.03) (Figure 3C), but had no significant effect on other events. Sensitivity analysis showed the results were stable in all events. The pooled results and sensitivity analysis of other events are displayed in Supplementary Figure S1.

In general, our study indicates that inclisiran has no significant effect on stroke or MACE but can reduce the risk of MI by 32%.

#### 3.4.2 Secondary outcomes

Inclisiran has significant lipid-lowering effects. In particular, it reduced the percentage change and absolute change of LDL-C by approximately 50% and 50 mg/dL. It also has a significant benefit in reducing PCSK9, TC, Apo B, and Non-HDL-C levels. The pooled effects of inclisiran on blood lipid and PCSK9 levels are shown in Table 2.

**TABLE 2:** Pooled effect of secondary outcomes^a^.

Secondary outcomes	Heterogeneity (I^2^, *p*-value)	Statistical model	WMD (95%CI)
Percentage Change in LDL-C From Baseline to Day 510 (%)	I^2^ = 72%, *p* = 0.03	REM	−53.98 (−58.30, −49.65)
Time-adjusted Percentage Change in LDL-C From Baseline After Day 90 and up to Day 540 (%)	I^2^ = 88%, *p* < 0.01	REM	−49.29 (−54.52, −44.07)
Absolute Change in LDL-C From Baseline to Day 510 (mg/dL)	I^2^ = 86%, *p* < 0.01	REM	−57.63 (−67.41, −47.86)
Time-adjusted Absolute Change in LDL-C From Baseline After Day 90 and up to Day 540 (mg/dL)	I^2^ = 89%, *p* < 0.01	REM	−54.52 (−62.09, −46.95)
Percentage Change in PCSK9 From Baseline to Day 510	I^2^ = 0%, *p* = 0.41	CEM	−79.67 (−81.89, −77.45)
Percentage Change in TC From Baseline to Day 510	I^2^ = 60%, *p* = 0.08	REM	−31.50 (−33.76, −29.23)
Percent Change in Apo B From Baseline to Day 510	I^2^ = 81%, *p* < 0.01	REM	−39.57 (−43.46, −35.69)
Percentage Change in Non-HDL-C From Baseline to Day 510	I^2^ = 61%, *p* = 0.08	REM	−44.63 (−47.71, −41.54)

^a^ In Table 2, REM, random effect model; CEM, common effect model; WMD, weighted mean difference; CI, confidence interval; LDL-C, low-density lipoprotein cholesterol; PCSK9, proprotein convertase subtilisin/kexin type 9; TC, total cholesterol; Apo B, Apolipoprotein B; Non-HDL-C, non-high-density lipoprotein cholesterol.

The safety populations were 1,833 in the inclisiran group and 1,822 in the placebo group. We performed a meta-analysis of TEAE, SAE, injection-site reaction, and some laboratory results in two groups. The pooled results showed that the inclisiran group had more significant injection-site reactions (*n* = 99) than the placebo group (*n* = 15) (RR = 6.56, 95%CI = 3.83–11.25), but they were mainly mild or moderate. There was no significant difference in other outcomes. The pooled results of safety outcomes are summarized in Table 3.

**TABLE 3:** Pooled effect of safety outcomes^a^.

No. of patients	Inclisiran (*n* = 1833)	Placebo (*n* = 1822)	Risk ratio (95%CI)	*p*-Value
TEAE				
Patients with≥1 TEAE	1430	1409	1.01 (0.97–1.05)	0.62
Patients with≥1 TEAE leading to discontinuation of trial intervention	45	35	1.28 (0.83–1.98)	0.27
SAE				
Patients with≥1 SAE	383	401	0.95 (0.84–1.08)	0.41
All-Cause Mortality	27	27	0.99 (0.59–1.69)	0.98
Cancer-related death	4	6	0.66 (0.19–2.34)	0.52
Cardiovascular cause	17	15	1.13 (0.56–2.25)	0.74
New worsening or compound cancer	44	49	0.89 (0.60–1.30)	0.58
Protocol-defined injection-site reaction				
Any event*	99	15	6.56 (3.83–11.25)	<0.001
Mild*	73	14	5.18 (2.94–9.15)	<0.001
Moderate*	26	1	25.84 (3.51–190.24)	0.001
Severe	0	0	-	-
Persistent	0	0	-	-
Laboratory results				
Liver function				
Alanine aminotransferase >3× ULN	7	5	1.39 (0.44–4.37)	0.57
Aspartate aminotransferase >3× ULN	8	10	0.80 (0.32–2.01)	0.63
Alkaline phosphatase >3× ULN	8	5	1.59 (0.52–4.85)	0.42
Bilirubin >2× ULN	14	14	0.99 (0.48–2.08)	0.99
Kidney function				
Creatinine >2 mg/dL	36	42	0.85 (0.55–1.32)	0.48
Muscle				
Creatine kinase >5× ULN	24	22	1.08 (0.61–1.93)	0.78
Hematology				
Platelet count <75,000 per mm^3^	1	2	0.50 (0.05–5.48)	0.57

*Has significant difference (*p* < 0.05).

^a^ In Table 3, TEAE, treatment-emergent adverse event; SAE, serious adverse event; ULN, upper limits of normal; CI, confidence interval.

#### 3.4.3 Descriptive analysis of ORION-5

ORION-5 was a two-part multicenter study to evaluate the safety, tolerability, and efficacy of inclisiran in subjects with HoFH. Subjects were randomized 2:1 to inclisiran: placebo. A total of 56 adults were enrolled, 34 being female (60.70%) and 48 being White (85.70%). There were 37 patients in the experimental group (34 completed part 1, 29 completed part 2) and 19 patients in the control group (all completed part 1, 18 completed part 2).

All subjects randomized into the study comprised the ITT population for outcomes analysis. The primary outcome was a percentage change in LDL-C from baseline to day 150; the result was 0.70 (95%CI = −14.03–15.44) in the inclisiran group and 2.39 (95%CI = −19.98–24.75) in the placebo group, with a mean difference of −1.68 (95%CI = −29.19–25.83, *p* = 0.98). The secondary outcomes included absolute and percentage changes of lipids (LDL-C, TC, HDL-C, etc.) and PCSK9 levels at follow-up time points. The results from Part 1 demonstrated that, other than significant differences observed in both the percentage change and absolute change of PCSK9 between the two groups, no significant differences in blood lipid. In part 2, only mean and standard deviation were reported, so we conducted an independent samples *t*-test and showed no significant differences (*p* > 0.05) in all lipid and PCSK9 levels.

In terms of safety conditions, for part 1, two SAEs (5.41%) and 12 other AEs (32.43%) occurred in the inclisiran group, and one SAE (5.26%) and six other AEs (31.58%) occurred in the placebo group. In part 2, 11 (20.75%) SAEs and 29 (54.72%) other AEs occurred, with one (1.89%) stroke and cerebrovascular accident event (carotid arteriosclerosis) and five (9.40%) MACE (angina unstable, carotid arteriosclerosis, cardiac failure, pulmonary edema, and sudden cardiac death). In addition, three subjects died in part 2.

## 4 Discussion

This systematic review and meta-analysis of 3,713 patients with ASCVD or at high risk of ASCVD showed that for those with a background of treatment with a maximum tolerated dose of statins or other lipid-regulation therapy, using inclisiran over 18 months can significantly reduce lipid and PCSK9 levels, with a 50% reduction in LDL-C and an 80% reduction in PCSK9. Nevertheless, the research findings revealed that while inclisiran was able to reduce the risk of MI by 32%, it did not demonstrate a significant correlation with the occurrence of stroke (be it compound stroke, ischaemic stroke, or hemorrhagic stroke) and MACE. 

In addition, compared with the placebo, inclisiran did not increase the overall occurrence of AEs but caused higher injection-site reactions. Most of the injection-site reactions were mild and moderate, and no severe and sustained reactions occurred.

Another study (Ray et al., 2022) also conducted a meta-analysis of the same trials but came to a different conclusion. Inclisiran was found to reduce the risk of MACE by 26% (relative risk [OR] = 0.74, 95%CI = 0.88–0.94) but was not associated with the occurrence risk of stroke (OR = 0.80, 95%CI = 0.50–1.27) and MI (OR = 0.86, 95%CI = 0.41–1.81). Through comparison, we found that the main reason was that the definitions of CVEs were different. Their definitions of stroke, MACE, and MI events are attached to Supplementary Table S5.

MACE, a common endpoint in cardiovascular studies, is a composite of clinical events, usually including endpoints reflecting safety and efficacy, which can reduce or eliminate the multiplicity problem of testing multiple endpoints. Additionally, accumulating evidence from individual endpoints to a composite endpoint can improve study power and reduce study size and trial duration (Huque et al., 2011). Due to the individual outcomes used to make this endpoint vary between studies, there was no standard definition of MACE. Therefore, the difference in the MACE definition among the studies and the unclear and incomplete reports make it impossible to compare, replicate, and summarize the study results (Bosco et al., 2021). Studies have shown that different definitions of MACE might lead to opposite results and conclusions (Kip et al., 2008). In addition, there was some variation in stroke from the statistic results of the most commonly used components of MACE, possibly due to differences in the definition of stroke, especially whether acute ischemic stroke with TIA, cerebral hemorrhage, or subarachnoid hemorrhage was included (Bosco et al., 2021).

In our study, we used SMQs to define CVEs. MedDRA is a medical dictionary for regulatory activities developed by The International Council for Harmonisation of Technical Requirements for Pharmaceuticals for Human Use. SMQs are formed by defining clusters of MedDRA terms that are highly relevant to medical conditions (Bill et al., 2012). Studies have confirmed that in the identification process of adverse events, SMQs can achieve higher sensitivity compared to PT and high-level term (HLT) (Pearson et al., 2009), so we adopted SMQs for the definition of CVEs. In addition, unlike the K et al. study, we analyzed both the overall event and its components. For example, stroke contains both hemorrhagic and ischemic events. Although it is still controversial whether lipid-regulating drugs cause hemorrhagic events, if inclisiran can reduce the occurrence of ischemic events and increase the occurrence of hemorrhagic events by lowering lipids, then mixing two events with opposite outcomes might have slashed the significance of the results. With that in mind, it makes sense that such effects could be avoided by analyzing and reporting hemorrhagic and ischemic events separately.

Nevertheless, even though the definitions of CVEs differed, the study by K et al. (Ray et al., 2022) and our study both show that inclisiran does not appear to contribute to the prevention of stroke.

A meta-analysis of other lipid-regulating treatments (non-inclisiran) (Lee et al., 2022) suggests that compared with less intensive LDL-C-lowering statin-based therapies (final mean LDL-C level = 119 mg/dL), more intensive therapies (final mean LDL-C level = 79 mg/dL) might be more favorable for stroke prevention (RR = 8.1% vs. 9.3%), especially for patients with evidence of atherosclerosis. In addition, lowering the LDL-C level was found to increase the risk of hemorrhagic stroke (RR = 1.46, 95%CI = 1.11–1.91). The mean follow-up duration of RCTs in this study was 4 years, while in our study, it was 1.5 years. Subgroup analysis of study duration based on the risk of compound stroke in the above study suggested there was no significant difference between the study durations of <3 years (RR = 0.92, 95%CI = 0.73–1.16) and ≥3 years (RR = 0.87, 95%CI = 0.79–0.96). Another study (Koskinas et al., 2018) found that compared to its significant LDL-C reduction, the reduction in the risk of CVEs with PCSK9 inhibitor treatment was within the expectations but increased after using Kaplan-Meier curves to extend the follow-up duration to be consistent with other RCTs. Since stroke is a chronic condition and prolonged follow-up duration may result in more cases and affect the outcome, we continue to believe that the length of follow-up influences outcomes. However, in the absence of long-term outcome data of inclisiran, this is merely a hypothesis. ORION-4, 2023 and VICTORION-2P, 2023 PREVENT were two large ongoing randomized, double-blind, placebo-controlled studies that investigated the impact of inclisiran on patients with ASCVD. They each expected to enroll 15,000 subjects with a follow-up duration of ≥5 years. Ischemic stroke was one of the primary outcomes. A larger number of subjects, longer follow-up duration, and more specific cardiovascular efficacy outcomes may clarify the association of inclisiran with stroke in ASCVD patients.

For hemorrhagic stroke, although the latest American College of Cardiology/American Heart Association guideline on the management of blood cholesterol (Grundy et al., 2019) states that it is not a statin-related AE, given conflicting literature data, the risk of hemorrhagic stroke might vary due to different lipid-regulating therapies (such as statins and PCSK9 inhibitors) or ethnicities (the association between lower LDL-C and a higher incidence of hemorrhagic stroke appears to be stronger in Asian people), it is unclear whether lower LDL-C is associated with a higher incidence of hemorrhagic stroke (Karagiannis et al., 2021).

ORION-5 indicated that compared with patients who were given the first two doses at a 3-month interval and then all further doses at 6-month intervals, there was no significant difference in the reduction of blood lipid and PCSK9 levels in those who were initially given inclisiran every 6 months. This suggests that inclisiran might be given semiannually from the beginning, rather than at 3-month intervals for the first and second doses. Further research is expected to verify this hypothesis.

We acknowledge that our study has some limitations. Firstly, the number and quality of the included studies were limited. All the included studies were assessed as having a high risk of bias, mainly because of detection bias and incomplete data reports. In addition, publication bias existed because of the small number of included trials, all of which were funded by medicine companies. Secondly, subgroup analysis was not conducted because the characteristics of included studies were similar and the data were insufficient in the number of CVEs in different populations. The countries and regions distribution (Table 1) of included clinical trials were different, and the impact was not explored in our study due to the limited data. Thirdly, the change in the definition of disease, such as stroke or MI, may impact the data, and the detection of stroke or MI may become more sensitive with the progression of time due to increased incidence. We cannot exclude the effects of definition and duration. Fourthly, we only updated the included studies at the end of the study; therefore, some studies published after this analysis might not be analyzed. Finally, as inclisiran is a new drug, there are few clinical studies and post-marketing studies with published results, so it is an objective fact that there is publication bias in this study. However, inclisiran has been approved by the European Union and FDA for the treatment of adults with HeFH or clinical ASCVD who require additional lowering of LDL-C and has already been used in the clinical setting. Clinical trials in other populations are also being steadily registered and are underway. As the results of clinical trials, post-marketing monitoring, and real-world studies are published, the efficacy and safety of inclisiran for specific populations will become clearer. We will continue to follow up and actively update the outcomes of the systematic review. We look forward to more updated and high-quality studies with larger samples in the future to validate the results and reach more convincing conclusions.

## 5 Conclusion

Lipid regulation is important for the prevention of stroke and cerebrovascular events in patients with ASCVD or at high risk of ASCVD. As a novel lipid-regulating drug, inclisiran has a significant effect in lowering blood lipids and PCSK9 levels. Our systematic review and meta-analysis showed that in patients with a history of treatment with a maximum tolerated dose of statins or other lipid-regulation therapy, using inclisiran is not beneficial for the prevention of stroke or cerebrovascular disease and MACE but is associated with a reduced risk of MI. However, due to the insufficient quantity and quality of literature and the non-standard definition of CVEs, further studies are expected to provide more details.

## Data Availability

The original contributions presented in the study are included in the article/Supplementary Material, further inquiries can be directed to the corresponding authors.

## References

[B1] Amarenco P. Labreuche J. Lavallée P. Touboul P. J. (2004). Statins in stroke prevention and carotid atherosclerosis: Systematic review and up-to-date meta-analysis. Stroke 35 (12), 2902–2909. 10.1161/01.STR.0000147965.52712.fa 15514180

[B2] Bill R. W. Liu Y. McInnes B. T. Melton G. B. Pedersen T. Pakhomov S. (2012). Evaluating semantic relatedness and similarity measures with Standardized MedDRA Queries. AMIA Annu. Symp. Proc. 2012, 43–50. 23304271 PMC3540472

[B3] Bosco E. Hsueh L. McConeghy K. W. Gravenstein S. Saade E. (2021). Major adverse cardiovascular event definitions used in observational analysis of administrative databases: A systematic review. BMC Med. Res. Methodol. 21 (1), 241. 10.1186/s12874-021-01440-5 34742250 PMC8571870

[B4] Cochrane (2022). Cochrane handbook for systematic reviews of interventions. Available At: https://training.cochrane.org/handbook/current (Accessed January 5, 2023).

[B5] Deng F. Mu C. Yang L. Li H. Xiang X. Li K. (2020). Carotid plaque magnetic resonance imaging and recurrent stroke risk: A systematic review and meta-analysis. Medicine 99 (13), e19377. 10.1097/MD.0000000000019377 32221065 PMC7220511

[B6] Fitzgerald K. Kallend D. Simon A. Bettencourt B. R. Strahs A. Clausen V. (2017). A highly durable RNAi therapeutic inhibitor of PCSK9. N. Engl. J. Med. 376 (18), 41–51. 10.1056/NEJMoa1609243 27959715 PMC5778873

[B7] Gangopadhyay S. Gore K. R. (2022). Advances in siRNA therapeutics and synergistic effect on siRNA activity using emerging dual ribose modifications. RNA Biol. 19 (1), 452–467. 10.1080/15476286.2022.2052641 35352626 PMC8973385

[B8] GBD 2019 Stroke Collaborators (2021). Global, regional, and national burden of stroke and its risk factors, 1990-2019: A systematic analysis for the global burden of disease study 2019. Lancet Neurol. 20 (10), 795–820. 10.1016/s1474-4422(21)00252-0 34487721 PMC8443449

[B9] Go G. W. Mani A. (2012). Low-density lipoprotein receptor (LDLR) family orchestrates cholesterol homeostasis. Yale J. Biol. Med. 85 (1), 19–28. 22461740 PMC3313535

[B10] Grundy S. M. Stone N. J. Bailey A. L. Beam C. Birtcher K. K. Blumenthal R. S. (2019). 2018 AHA/ACC/AACVPR/AAPA/ABC/ACPM/ADA/AGS/APhA/ASPC/NLA/PCNA guideline on the management of blood cholesterol: A report of the American College of Cardiology/American heart association task force on clinical Practice guidelines. Circulation 139 (25), e1082–e1143. 10.1161/CIR.0000000000000625 30586774 PMC7403606

[B11] Hackam D. G. Hegele R. A. (2022). Lipid-modifying therapies and stroke prevention. Curr. Neurol. Neurosci. Rep. 22 (7), 375–382. 10.1007/s11910-022-01197-4 35554824

[B12] Holmes M. V. Millwood I. Y. Kartsonaki C. Hill M. R. Bennett D. A. Boxall R. (2018). Lipids, lipoproteins, and metabolites and risk of myocardial infarction and stroke. J. Am. Coll. Cardiol. 71 (6), 620–632. 10.1016/j.jacc.2017.12.006 29420958 PMC5811927

[B13] Huque M. F. Alosh M. Bhore R. (2011). Addressing multiplicity issues of a composite endpoint and its components in clinical trials. J. Biopharm. statistics 21 (4), 610–634. 10.1080/10543406.2011.551327 21516560

[B14] Karagiannis A. D. Mehta A. Dhindsa D. S. Virani S. S. Orringer C. E. Blumenthal R. S. (2021). How low is safe? The frontier of very low (<30 mg/dL) LDL cholesterol. Eur. Heart J. 42 (22), 2154–2169. 10.1093/eurheartj/ehaa1080 33463677 PMC12784414

[B15] Khan S. U. Yedlapati S. H. Lone A. N. Hao Q. Guyatt G. Delvaux N. (2022). PCSK9 inhibitors and ezetimibe with or without statin therapy for cardiovascular risk reduction: A systematic review and network meta-analysis. BMJ Clin. Res. ed. 377, e069116. 10.1136/bmj-2021-069116 35508321

[B16] Khatana C. Saini N. K. Chakrabarti S. Saini V. Sharma A. Saini R. V. (2020). Mechanistic insights into the oxidized low-density lipoprotein-induced atherosclerosis. Oxidative Med. Cell. Longev. 2020, 5245308. 10.1155/2020/5245308 PMC7512065 33014272

[B17] Khvorova A. (2017). Oligonucleotide therapeutics - a new class of cholesterol-lowering drugs. N. Engl. J. Med. 376 (1), 4–7. 10.1056/NEJMp1614154 28052224

[B18] Kip K. E. Hollabaugh K. Marroquin O. C. Williams D. O. (2008). The problem with composite end points in cardiovascular studies: The story of major adverse cardiac events and percutaneous coronary intervention. J. Am. Coll. Cardiol. 51 (7), 701–707. 10.1016/j.jacc.2007.10.034 18279733

[B19] Kleindorfer D. O. Towfighi A. Chaturvedi S. Cockroft K. M. Gutierrez J. Lombardi-Hill D. (2021). 2021 guideline for the prevention of stroke in patients with stroke and transient ischemic attack: A guideline from the American heart association/American stroke association. Stroke 52 (7), e364–e467. 10.1161/str.0000000000000375 34024117

[B20] Koskinas K. C. Siontis G. C. M. Piccolo R. Mavridis D. Räber L. Mach F. (2018). Effect of statins and non-statin LDL-lowering medications on cardiovascular outcomes in secondary prevention: A meta-analysis of randomized trials. Eur. heart J. 39 (14), 1172–1180. 10.1093/eurheartj/ehx566 29069377

[B21] Kuriakose D. Xiao Z. (2020). Pathophysiology and treatment of stroke: Present status and future perspectives. Int. J. Mol. Sci. 21 (20), 7609. 10.3390/ijms21207609 33076218 PMC7589849

[B22] Lee M. Cheng C. Y. Wu Y. L. Lee J. D. Hsu C. Y. Ovbiagele B. (2022). Association between intensity of low-density lipoprotein cholesterol reduction with statin-based therapies and secondary stroke prevention: A meta-analysis of randomized clinical trials. JAMA Neurol. 79 (4), 349–358. 10.1001/jamaneurol.2021.5578 35188949 PMC8861901

[B23] Medeiros G. C. Roy D. Kontos N. Beach S. R. (2020). Post-stroke depression: A 2020 updated review. General Hosp. Psychiatry 66, 70–80. 10.1016/j.genhosppsych.2020.06.011 32717644

[B24] Moustafa B. Testai F. D. (2021). Efficacy and safety of PCSK9 inhibitors in stroke prevention. J. stroke Cerebrovasc. Dis. 30 (11), 106057. 10.1016/j.jstrokecerebrovasdis.2021.106057 34450482

[B25] ORION-4 (2023). Study description of ORION-4. Available At: https://clinicaltrials.gov/ct2/show/NCT03705234?term=ORION-4&draw=2&rank=1 (Accessed January 5, 2023).

[B26] ORION-5 (2023). Data from clinical trials of ORION-5. Available At: https://clinicaltrials.gov/ct2/show/NCT03851705?term=ORION-5&draw=2&rank=1 (Accessed January 5, 2023).

[B27] Page M. J. McKenzie J. E. Bossuyt P. M. Boutron I. Hoffmann T. C. Mulrow C. D. (2021). The PRISMA 2020 statement: An updated guideline for reporting systematic reviews. Bmj 372, n71. 10.1136/bmj.n71 33782057 PMC8005924

[B28] Pasi M. Poggesi A. Salvadori E. Pantoni L. (2012). Post-stroke dementia and cognitive impairment. Front. Neurol. Neurosci. 30, 65–69. 10.1159/000333412 22377866

[B29] Pearson R. K. Hauben M. Goldsmith D. I. Gould A. L. Madigan D. O'Hara D. J. (2009). Influence of the MedDRA ® hierarchy on pharmacovigilance data mining results. Int. J. Med. Inf. 78 (12), e97–e103. 10.1016/j.ijmedinf.2009.01.001 19230751

[B30] Raal F. J. Kallend D. Ray K. K. Turner T. Koenig W. Wright R. S. (2020). Inclisiran for the treatment of heterozygous familial hypercholesterolemia. N. Engl. J. Med. 382 (16), 1520–1530. 10.1056/NEJMoa1913805 32197277

[B31] Ray K. K. Raal F. J. Kallend D. G. Jaros M. J. Koenig W. Leiter L. A. (2022). Inclisiran and cardiovascular events: A patient-level analysis of phase III trials. Eur. heart J. 44, 129–138. 10.1093/eurheartj/ehac594 PMC9825807 36331326

[B32] Ray K. K. Wright R. S. Kallend D. Koenig W. Leiter L. A. Raal F. J. (2020). Two phase 3 trials of inclisiran in patients with elevated LDL cholesterol. N. Engl. J. Med. 382 (16), 1507–1519. 10.1056/NEJMoa1912387 32187462

[B33] Riley R. D. Moons K. G. M. Snell K. I. E. Ensor J. Hooft L. Altman D. G. (2019). A guide to systematic review and meta-analysis of prognostic factor studies. BMJ 364, 4597. 10.1136/bmj.k4597 30700442

[B34] Rogers J. Baker M. (2020). Understanding the most commonly billed diagnoses in primary care: Atherosclerotic cardiovascular disease. Nurse Pract. 45 (7), 35–41. 10.1097/01.NPR.0000669136.88720.65 32568795

[B35] Shamseer L. Moher D. Clarke M. Ghersi D. Liberati A. Petticrew M. (2015). Preferred reporting items for systematic review and meta-analysis protocols (PRISMA-P) 2015: Elaboration and explanation. Bmj 354, g7647. 10.1136/bmj.g7647 25555855

[B36] Soffer D. Stoekenbroek R. Plakogiannis R. (2022). Small interfering ribonucleic acid for cholesterol lowering - inclisiran: Inclisiran for cholesterol lowering. J. Clin. Lipidol. 16 (5), 574–582. 10.1016/j.jacl.2022.06.009 35909047

[B37] Springer A. D. Dowdy S. F. (2018). GalNAc-siRNA conjugates: Leading the way for delivery of RNAi therapeutics. Nucleic acid. Ther. 28 (3), 109–118. 10.1089/nat.2018.0736 29792572 PMC5994659

[B38] VICTORION-2P (2023). Study description of VICTORION-2P. Available At: https://clinicaltrials.gov/ct2/show/NCT05030428?term=VICTORION-2+Prevent&draw=2&rank=1 (Accessed January 5, 2023).

[B39] Xing Y. An Z. Yu N. Zhao W. Ning X. Wang J. (2016). Low density lipoprotein cholesterol and the outcome of acute ischemic stroke: Results of a large hospital-based study. Eur. Neurol. 76 (5-6), 195–201. 10.1159/000450604 27705971

[B40] Yuan B. B. Luo G. G. Gao J. X. Qiao J. Yang J. B. Huo K. (2015). Variance of serum lipid levels in stroke subtypes. Clin. Lab. 61 (10), 1509–1514. 10.7754/clin.lab.2015.150118 26642713

