# Supplementary material for: Efficacy and safety of inclisiran in stroke or cerebrovascular disease prevention: a systematic review and meta-analysis of randomized controlled trials
Source: Front Pharmacol. 2023 Jun 13;14:1158274. doi: 10.3389/fphar.2023.1158274 (PMC10299829; doi:10.3389/fphar.2023.1158274)
Supplement: Supplementary file 1 [file DataSheet1.docx]

**Supplementary Table 1.** The PRISMA 2020 checklist(Page et al., 2021)

| **Section and Topic** | **Item#** | **Checklist item** | **Location** |
| --- | --- | --- | --- |
| **TITLE** | | |  |
| Title | 1 | Identify the report as a systematic review. | Page 1; Line 1-2 |
| **ABSTRACT** | | |  |
| Abstract | 2 | See the PRISMA 2020 for Abstracts checklist. | Page 1; Line 12-33 |
| **INTRODUCTION** | | |  |
| Rationale | 3 | Describe the rationale for the review in the context of existing knowledge. | Page 2-3; Line 35-104 |
| Objectives | 4 | Provide an explicit statement of the objective(s) or question(s) the review addresses. | Page 3; Line 102-104 |
| **METHODS** | | |  |
| Eligibility criteria | 5 | Specify the inclusion and exclusion criteria for the review and how studies were grouped for the syntheses. | Page 4; Line 124-136 |
| Information sources | 6 | Specify all databases, registers, websites, organisations, reference lists and other sources searched or consulted to identify studies. Specify the date when each source was last searched or consulted. | Page 3-4; Line 111-123 |
| Search strategy | 7 | Present the full search strategies for all databases, registers and websites, including any filters and limits used. | Supplementary Tab. 1 |
| Selection process | 8 | Specify the methods used to decide whether a study met the inclusion criteria of the review, including how many reviewers screened each record and each report retrieved, whether they worked independently, and if applicable, details of automation tools used in the process. | Page 4; Line 137-142 |
| Data collection process | 9 | Specify the methods used to collect data from reports, including how many reviewers collected data from each report, whether they worked independently, any processes for obtaining or confirming data from study investigators, and if applicable, details of automation tools used in the process. | Page 4; Line 144-150 |
| Data items | 10a | List and define all outcomes for which data were sought. Specify whether all results that were compatible with each outcome domain in each study were sought (e.g. for all measures, time points, analyses), and if not, the methods used to decide which results to collect. | Page 4; Line 151-154 |
|  | 10b | List and define all other variables for which data were sought (e.g. participant and intervention characteristics, funding sources). Describe any assumptions made about any missing or unclear information. | Page 4; Line 155-163 & Supplementary Tab. 3, Tab. 4 |
| Study risk of bias assessment | 11 | Specify the methods used to assess risk of bias in the included studies, including details of the tool(s) used, how many reviewers assessed each study and whether they worked independently, and if applicable, details of automation tools used in the process. | Page 5; Line 165-177 |
| Effect measures | 12 | Specify for each outcome the effect measure(s) (e.g. risk ratio, mean difference) used in the synthesis or presentation of results. | Page 5; Line 184-188 |
| Synthesis methods | 13a | Describe the processes used to decide which studies were eligible for each synthesis (e.g. tabulating the study intervention characteristics and comparing against the planned groups for each synthesis (item #5)). | Page 5-6; Line 206-225 & Tab. 1 |
|  | 13b | Describe any methods required to prepare the data for presentation or synthesis, such as handling of missing summary statistics, or data conversions. | Not applicable |
|  | 13c | Describe any methods used to tabulate or visually display results of individual studies and syntheses. | Page 5; Line 188-189 |
|  | 13d | Describe any methods used to synthesize results and provide a rationale for the choice(s). If meta-analysis was performed, describe the model(s), method(s) to identify the presence and extent of statistical heterogeneity, and software package(s) used. | Page 5; Line 179-184 |
|  | 13e | Describe any methods used to explore possible causes of heterogeneity among study results (e.g. subgroup analysis, meta-regression). | Not applicable |
|  | 13f | Describe any sensitivity analyses conducted to assess robustness of the synthesized results. | Page 5; Line 189-191 |
| Reporting bias assessment | 14 | Describe any methods used to assess risk of bias due to missing results in a synthesis (arising from reporting biases). | Page 5; Line 165-177 |
| Certainty assessment | 15 | Describe any methods used to assess certainty (or confidence) in the body of evidence for an outcome. | Not applicable |
| **RESULTS** | | |  |
| Study selection | 16a | Describe the results of the search and selection process, from the number of records identified in the search to the number of studies included in the review, ideally using a flow diagram. | Page 5; Line 195-204 & Fig. 1 |
|  | 16b | Cite studies that might appear to meet the inclusion criteria, but which were excluded, and explain why they were excluded. | Fig. 1 |
| Study characteristics | 17 | Cite each included study and present its characteristics. | Page 6; Line 206-225 |
| Risk of bias in studies | 18 | Present assessments of risk of bias for each included study. | Page 6-7; Line 227-255 & Fig. 2 |
| Results of individual studies | 19 | For all outcomes, present, for each study: (a) summary statistics for each group (where appropriate) and (b) an effect estimate and its precision (e.g. confidence/credible interval), ideally using structured tables or plots. | Page 7-8; Line 256-298 & Tab. 2, Tab. 3, Fig. 3 & Supplementary Fig.1 |
| Results of syntheses | 20a | For each synthesis, briefly summarise the characteristics and risk of bias among contributing studies. |  |
|  | 20b | Present results of all statistical syntheses conducted. If meta-analysis was done, present for each the summary estimate and its precision (e.g. confidence/credible interval) and measures of statistical heterogeneity. If comparing groups, describe the direction of the effect. |  |
|  | 20c | Present results of all investigations of possible causes of heterogeneity among study results. |  |
|  | 20d | Present results of all sensitivity analyses conducted to assess the robustness of the synthesized results. |  |
| Reporting biases | 21 | Present assessments of risk of bias due to missing results (arising from reporting biases) for each synthesis assessed. | Not applicable |
| Certainty of evidence | 22 | Present assessments of certainty (or confidence) in the body of evidence for each outcome assessed. | Not applicable |
| **DISCUSSION** | | |  |
| Discussion | 23a | Provide a general interpretation of the results in the context of other evidence. | Page 8-10; Line 321-386 |
|  | 23b | Discuss any limitations of the evidence included in the review. | Page 10; Line 387-396 |
|  | 23c | Discuss any limitations of the review processes used. | Page 10; Line 396-398 |
|  | 23d | Discuss implications of the results for practice, policy, and future research. | Page 10; Line 387-400 |
| **OTHER INFORMATION** | | |  |
| Registration and protocol | 24a | Provide registration information for the review, including register name and registration number, or state that the review was not registered. | Page 3; Line 106-109 |
|  | 24b | Indicate where the review protocol can be accessed, or state that a protocol was not prepared. | - |
|  | 24c | Describe and explain any amendments to information provided at registration or in the protocol. | - |
| Support | 25 | Describe sources of financial or non-financial support for the review, and the role of the funders or sponsors in the review. | Page 10; Line 419-421 |
| Competing interests | 26 | Declare any competing interests of review authors. | Page 10; Line 410-412 |
| Availability of data, code and other materials | 27 | Report which of the following are publicly available and where they can be found: template data collection forms; data extracted from included studies; data used for all analyses; analytic code; any other materials used in the review. | Page 10-11; Line 422-424 |

**Supplementary Table 2.** Search strategy (a) Pubmed; (b) Embase; (c) CENTRAL; (d) Web of science; (e) ClinicalTrials.gov; (f) WHO ICTRP

**(a) Pubmed**

| **Serial** | **Search strategy** | **17 Oct. 2022** | **6 Mar. 2023** |
| --- | --- | --- | --- |
| #1 | (((((((((((((((((("Arterial Occlusive Diseases"[Mesh]) OR "Arteriolosclerosis"[Mesh]) OR "Atherosclerosis"[Mesh]) OR "Coronary Artery Disease"[Mesh]) OR "Peripheral Arterial Disease"[Mesh]) OR "Coronary Disease"[Mesh]) OR "Acute Coronary Syndrome"[Mesh]) OR "Cardiovascular Diseases"[Mesh]) OR "Heart Disease Risk Factors"[Mesh]) OR "Angina, Unstable"[Mesh]) OR "Myocardial Infarction"[Mesh]) OR "Stroke"[Mesh]) OR "Cerebrovascular Disorders"[Mesh]) OR "Peripheral Arterial Disease"[Mesh]) OR "Ischemic Attack, Transient"[Mesh]) OR "percutaneous coronary intervention"[Mesh]) OR "stents"[Mesh]) OR "Coronary Artery Bypass"[Mesh]) | 2,703,492 | 2,737,903 |
| #2 | (Arterial Occlusive Disease*[Title/Abstract]) OR (Disease*, Arterial Occlusive[Title/Abstract]) OR (Occlusive Disease*, Arterial[Title/Abstract]) OR (Arterial Obstructive* Disease[Title/Abstract]) OR (Disease*, Arterial Obstructive[Title/Abstract]) OR (Obstructive Disease*, Arterial[Title/Abstract]) OR (Arterioloscleroses[Title/Abstract]) OR (Atheroscleroses[Title/Abstract]) OR (Atherogenesis[Title/Abstract]) OR (Artery Disease*, Coronary[Title/Abstract]) OR (Coronary Artery Disease*[Title/Abstract]) OR (Left Main Disease*[Title/Abstract]) OR (Left Main Coronary Disease[Title/Abstract]) OR (Coronary Arteriosclerosis[Title/Abstract]) OR (Arterioscleroses, Coronary[Title/Abstract]) OR (Coronary Arterioscleroses[Title/Abstract]) OR (Atherosclerosis, Coronary[Title/Abstract]) OR (Atheroscleroses, Coronary[Title/Abstract]) OR (Coronary Atheroscleroses[Title/Abstract]) OR (Coronary Atherosclerosis[Title/Abstract]) OR (Arteriosclerosis, Coronary[Title/Abstract]) OR (Arterial Disease*, Peripheral[Title/Abstract]) OR (Disease*, Peripheral Arterial[Title/Abstract]) OR (Peripheral Artery Disease*[Title/Abstract]) OR (Artery Disease*, Peripheral[Title/Abstract]) OR (Disease*, Peripheral Artery[Title/Abstract]) OR (Peripheral Artery Diseases[Title/Abstract]) OR (Coronary Diseases[Title/Abstract]) OR (Disease*, Coronary[Title/Abstract]) OR (Coronary Heart Disease*[Title/Abstract]) OR (Disease*, Coronary Heart[Title/Abstract]) OR (Heart Disease*, Coronary[Title/Abstract]) OR (Acute Coronary Syndromes[Title/Abstract]) OR (Coronary Syndrome*, Acute[Title/Abstract]) OR (Syndrome*, Acute Coronary[Title/Abstract]) OR (Cardiovascular Disease[Title/Abstract]) OR (Disease*, Cardiovascular[Title/Abstract]) OR (Cardiovascular Risk*[Title/Abstract]) OR (Risk Factor, Cardiovascular[Title/Abstract]) OR (Risk Factors for Heart Disease[Title/Abstract]) OR (Risk Factors for Cardiovascular Disease[Title/Abstract]) OR (Risk Score, Cardiovascular[Title/Abstract]) OR (Risk, Cardiovascular[Title/Abstract]) OR (Risk, Residual Cardiovascular[Title/Abstract]) OR (Anginas, Unstable[Title/Abstract]) OR (Unstable Angina*[Title/Abstract]) OR (Angina Pectori*, Unstable[Title/Abstract]) OR (Unstable Angina Pectori*[Title/Abstract]) OR (Angina at Rest[Title/Abstract]) OR (Angina*, Preinfarction[Title/Abstract]) OR (Preinfarction Angina*[Title/Abstract]) OR (Myocardial Preinfarction Syndrome*[Title/Abstract]) OR (Preinfarction Syndrome*, Myocardial[Title/Abstract]) OR (Syndrome*, Myocardial Preinfarction[Title/Abstract]) OR (Infarction*, Myocardial[Title/Abstract]) OR (Cardiovascular Stroke*[Title/Abstract]) OR (Stroke*, Cardiovascular[Title/Abstract]) OR (Myocardial Infarct*[Title/Abstract]) OR (Infarct*, Myocardial[Title/Abstract]) OR (Heart Attack*[Title/Abstract]) OR (Strokes[Title/Abstract]) OR (Cerebrovascular Accident*[Title/Abstract]) OR (CVA*[Title/Abstract]) OR (Cerebrovascular Apoplexy[Title/Abstract]) OR (Apoplexy, Cerebrovascular[Title/Abstract]) OR (Vascular Accident, Brain[Title/Abstract]) OR (Brain Vascular Accident*[Title/Abstract]) OR (Vascular Accidents, Brain[Title/Abstract]) OR (Cerebrovascular Stroke*[Title/Abstract]) OR (Stroke*, Cerebrovascular[Title/Abstract]) OR (Apoplexy[Title/Abstract]) OR (Cerebral Stroke*[Title/Abstract]) OR (Stroke*, Cerebral[Title/Abstract]) OR (Stroke*, Acute[Title/Abstract]) OR (Acute Stroke*[Title/Abstract]) OR (Cerebrovascular Accident*, Acute[Title/Abstract]) OR (Acute Cerebrovascular Accident*[Title/Abstract]) OR (Cerebrovascular Disorder[Title/Abstract]) OR (Cerebrovascular Disease*[Title/Abstract]) OR (Disease*, Cerebrovascular[Title/Abstract]) OR (Intracranial Vascular Disease*[Title/Abstract]) OR (Vascular Disease*, Intracranial[Title/Abstract]) OR (Brain Vascular Disorder*[Title/Abstract]) OR (Vascular Disorder*, Brain[Title/Abstract]) OR (Intracranial Vascular Disorder*[Title/Abstract]) OR (Vascular Disorder*, Intracranial[Title/Abstract]) OR (Cerebrovascular Insufficienc*[Title/Abstract]) OR (Insufficienc*, Cerebrovascular[Title/Abstract]) OR (Cerebrovascular Occlusion*[Title/Abstract]) OR (Occlusion*, Cerebrovascular[Title/Abstract]) OR (Arterial Disease*, Peripheral[Title/Abstract]) OR (Disease*, Peripheral Arterial[Title/Abstract]) OR (Peripheral Arterial Diseases[Title/Abstract]) OR (Peripheral Artery Disease*[Title/Abstract]) OR (Artery Disease*, Peripheral[Title/Abstract]) OR (Disease*, Peripheral Artery[Title/Abstract]) OR (TIA*[Title/Abstract]) OR (Transient Ischemic Attack*[Title/Abstract]) OR (Attack*, Transient Ischemic[Title/Abstract]) OR (Ischemic Attacks, Transient[Title/Abstract]) OR (Cerebral Ischemia*, Transient[Title/Abstract]) OR (Ischemia*, Transient Cerebral[Title/Abstract]) OR (Transient Cerebral Ischemia*[Title/Abstract]) OR (Brain Stem Ischemia, Transient[Title/Abstract]) OR (Brainstem Ischemia*, Transient[Title/Abstract]) OR (Ischemia*, Transient Brainstem[Title/Abstract]) OR (Transient Brainstem Ischemia[Title/Abstract]) OR (Coronary Intervention*, Percutaneous[Title/Abstract]) OR (Intervention*, Percutaneous Coronary[Title/Abstract]) OR (Percutaneous Coronary Intervention*[Title/Abstract]) OR (Percutaneous Coronary Revascularization*[Title/Abstract]) OR (Coronary Revascularization*, Percutaneous[Title/Abstract]) OR (Revascularization*, Percutaneous Coronary[Title/Abstract]) OR (stent[Title/Abstract]) OR (Artery Bypass*, Coronary[Title/Abstract]) OR (Bypass*, Coronary Artery[Title/Abstract]) OR (Coronary Artery Bypass*[Title/Abstract]) OR (Aortocoronary Bypass*[Title/Abstract]) OR (Bypass*, Aortocoronary[Title/Abstract])(ASCVD[Title/Abstract]) OR (CHD[Title/Abstract]) OR (PAD[Title/Abstract]) OR (CVA[Title/Abstract]) OR (MI[Title/Abstract]) OR (CAD[Title/Abstract]) OR (TIA[Title/Abstract]) OR (CABG[Title/Abstract]) | 191,587 | 196,936 |
| #3 | #1 OR #2 | 2,786,555 | 2,823,734 |
| #4 | (ALN PCSsc[Title/Abstract]) OR (ALN 60212[Title/Abstract]) OR (PCSK9si KJX 839[Title/Abstract]) OR (Inclisiran[Title/Abstract]) OR (small interfering RNA[Title/Abstract]) OR (RNAi[Title/Abstract]) OR (SiRNA[Title/Abstract]) OR (RNA, Small Interfering[Title/Abstract]) | 106,582 | 108,842 |
| #5 | (randomized controlled trial[pt] OR controlled clinical trial[pt] OR randomized[tiab] OR placebo[tiab] OR drug therapy[sh] OR randomly[tiab] OR trial[tiab] OR groups[tiab]) NOT (animals[mh] NOT humans[mh]) | 4,848,788 | 4,953,672 |
| #6 | #3 AND #4 AND #5 | **600** | **631** |

**(b) Embase**

| **Serial** | **Search strategy** | **17 Oct. 2022** | **6 Mar. 2023** |
| --- | --- | --- | --- |
| #1 | 'peripheral occlusive artery disease'/exp OR 'arteriolosclerosis'/exp OR 'atherosclerosis'/exp OR 'coronary artery disease'/exp OR 'acute coronary syndrome'/exp OR 'cardiovascular disease'/exp OR 'heart disease risk factor'/exp OR 'unstable angina pectoris'/exp OR 'heart infarction'/exp OR 'cerebrovascular accident'/exp OR 'cerebrovascular disease'/exp OR 'transient ischemic attack'/exp OR 'percutaneous coronary intervention'/exp OR 'stent'/exp OR 'coronary artery bypass graft'/exp | 5,296,699 | 5,408,505 |
| #2 | 'arterial disease, peripheral occlusive':ab,ti,kw OR 'arterial obliteration':ab,ti,kw OR 'arterial obliterative disease':ab,ti,kw OR 'arterial occlusive diseases':ab,ti,kw OR 'arteriosclerosis obliterans':ab,ti,kw OR 'artery chronic occlusive disease':ab,ti,kw OR 'artery obliterative disease':ab,ti,kw OR 'artery occlusive disease':ab,ti,kw OR 'artery peripheral occlusion':ab,ti,kw OR 'atheriosclerotic occlusion':ab,ti,kw OR 'atherosclerosis obliterans':ab,ti,kw OR 'atherosclerotic occlusion':ab,ti,kw OR 'atherosclerotic peripheral arterial insufficiency':ab,ti,kw OR 'chronic arterial occlusion disease':ab,ti,kw OR 'chronic artery obstruction':ab,ti,kw OR 'chronic artery occlusion':ab,ti,kw OR 'chronic occlusion, artery':ab,ti,kw OR 'obliterating arteriosclerosis':ab,ti,kw OR 'obliterating atherosclerosis':ab,ti,kw OR 'obliterative arterial disease':ab,ti,kw OR 'obliterative arteriosclerosis':ab,ti,kw OR 'obliterative artery disease':ab,ti,kw OR 'obliterative atherosclerosis':ab,ti,kw OR 'obliterative vascular disease':ab,ti,kw OR 'obstructive arterial disease':ab,ti,kw OR 'obstructive artery disease':ab,ti,kw OR 'obstructive vascular disease':ab,ti,kw OR 'occlusive arterial disease':ab,ti,kw OR 'occlusive artery disease':ab,ti,kw OR 'occlusive vascular disease':ab,ti,kw OR 'pad (peripheral arterial disease)':ab,ti,kw OR 'paod':ab,ti,kw OR 'peripheral arterial disease':ab,ti,kw OR 'peripheral arterial diseases':ab,ti,kw OR 'peripheral arterial obstructive disease':ab,ti,kw OR 'peripheral arterial occlusive disease':ab,ti,kw OR 'peripheral arterial occlusive diseases':ab,ti,kw OR 'peripheral artery disease':ab,ti,kw OR 'peripheral artery obstruction':ab,ti,kw OR 'peripheral artery obstructive disease':ab,ti,kw OR 'peripheral artery occlusion':ab,ti,kw OR 'peripheral artery occlusive disease':ab,ti,kw OR 'peripheral obliterative arterial disease':ab,ti,kw OR 'peripheral obliterative vascular disease':ab,ti,kw OR 'peripheral obstructive artery disease':ab,ti,kw OR 'peripheral occlusive arterial disease':ab,ti,kw OR 'peripheral occlusive disease':ab,ti,kw OR 'vascular occlusive disease':ab,ti,kw OR 'arteriolar sclerosis':ab,ti,kw OR 'arteriolo-sclerosis':ab,ti,kw OR 'arteriolosclerotic disease':ab,ti,kw OR 'arteriolosclerotic small vessel disease':ab,ti,kw OR 'arteriolosis':ab,ti,kw OR 'atheriosclerotic disease':ab,ti,kw OR 'athero-sclerosis':ab,ti,kw OR 'atheromatous sclerosis':ab,ti,kw OR 'atherosclerosis grading':ab,ti,kw OR 'atherosclerotic disease':ab,ti,kw OR 'atherosclerotic disorder':ab,ti,kw OR 'atherosclerotic vascular disease':ab,ti,kw OR 'atherosclerotic vascular disorder':ab,ti,kw OR 'coronary disease':ab,ti,kw OR 'multivessel coronary artery disease':ab,ti,kw OR 'acute coronary syndromes':ab,ti,kw OR 'angiocardiopathy':ab,ti,kw OR 'angiocardiovascular disease':ab,ti,kw OR 'cardiovascular complication':ab,ti,kw OR 'cardiovascular diseases':ab,ti,kw OR 'cardiovascular disorder':ab,ti,kw OR 'cardiovascular disturbance':ab,ti,kw OR 'cardiovascular lesion':ab,ti,kw OR 'cardiovascular syndrome':ab,ti,kw OR 'cardiovascular vegetative disorder':ab,ti,kw OR 'complication, cardiovascular':ab,ti,kw OR 'disease, cardiovascular':ab,ti,kw OR 'major adverse cardiovascular event':ab,ti,kw OR 'cardiac disease risk factors':ab,ti,kw OR 'heart disease risk factors':ab,ti,kw OR 'risk factor for cardiac disease':ab,ti,kw OR 'risk factor for heart disease':ab,ti,kw OR 'risk factors for cardiac disease':ab,ti,kw OR 'risk factors for heart disease':ab,ti,kw OR 'angina pectoris, unstable':ab,ti,kw OR 'angina, unstable':ab,ti,kw OR 'unstable angina':ab,ti,kw OR 'cardiac infarct':ab,ti,kw OR 'cardiac infarction':ab,ti,kw OR 'cardial infarct':ab,ti,kw OR 'heart attack':ab,ti,kw OR 'heart infarct':ab,ti,kw OR 'heart micro infarction':ab,ti,kw OR 'heart muscle infarction':ab,ti,kw OR 'infarction, heart':ab,ti,kw OR 'myocardial infarct':ab,ti,kw OR 'myocardial infarction':ab,ti,kw OR 'myocardium infarct':ab,ti,kw OR 'myocardium infarction':ab,ti,kw OR 'premonitory infarction sign':ab,ti,kw OR 'second heart attack':ab,ti,kw OR 'subendocardial infarction':ab,ti,kw OR 'transmural cardiac infarction':ab,ti,kw OR 'transmural heart infarction':ab,ti,kw OR 'transmural infarction, heart':ab,ti,kw OR 'accident, cerebrovascular':ab,ti,kw OR 'acute cerebrovascular lesion':ab,ti,kw OR 'acute focal cerebral vasculopathy':ab,ti,kw OR 'acute stroke':ab,ti,kw OR 'apoplectic stroke':ab,ti,kw OR 'apoplexia':ab,ti,kw OR 'apoplexy':ab,ti,kw OR 'blood flow disturbance, brain':ab,ti,kw OR 'brain accident':ab,ti,kw OR 'brain attack':ab,ti,kw OR 'brain blood flow disturbance':ab,ti,kw OR 'brain insult':ab,ti,kw OR 'brain insultus':ab,ti,kw OR 'brain vascular accident':ab,ti,kw OR 'cerebral apoplexia':ab,ti,kw OR 'cerebral insult':ab,ti,kw OR 'cerebral stroke':ab,ti,kw OR 'cerebral vascular accident':ab,ti,kw OR 'cerebral vascular insufficiency':ab,ti,kw OR 'cerebro vascular accident':ab,ti,kw OR 'cerebrovascular arrest':ab,ti,kw OR 'cerebrovascular failure':ab,ti,kw OR 'cerebrovascular injury':ab,ti,kw OR 'cerebrovascular insufficiency':ab,ti,kw OR 'cerebrovascular insult':ab,ti,kw OR 'cerebrum vascular accident':ab,ti,kw OR 'cryptogenic stroke':ab,ti,kw OR 'insultus cerebralis':ab,ti,kw OR 'ischaemic seizure':ab,ti,kw OR 'ischemic seizure':ab,ti,kw OR 'stroke':ab,ti,kw OR 'thrombotic stroke':ab,ti,kw OR 'brain angiopathy':ab,ti,kw OR 'brain circulation failure':ab,ti,kw OR 'brain vascular disease':ab,ti,kw OR 'brain vasculopathy':ab,ti,kw OR 'cerebral angiopathy':ab,ti,kw OR 'cerebral small vessel disease':ab,ti,kw OR 'cerebral small vessel diseases':ab,ti,kw OR 'cerebral vascular disease':ab,ti,kw OR 'cerebral vascular disorder':ab,ti,kw OR 'cerebral vascular disturbance':ab,ti,kw OR 'cerebral vascular lesion':ab,ti,kw OR 'cerebral vasculopathy':ab,ti,kw OR 'cerebro-vascular damage':ab,ti,kw OR 'cerebro-vascular disease':ab,ti,kw OR 'cerebro-vascular disorder':ab,ti,kw OR 'cerebro-vascular disturbance':ab,ti,kw OR 'cerebro-vascular lesion':ab,ti,kw OR 'cerebro-vascular pathology':ab,ti,kw OR 'cerebro-vascular syndrome':ab,ti,kw OR 'cerebro-vasculopathy':ab,ti,kw OR 'cerebroangiopathy':ab,ti,kw OR 'cerebrovascular damage':ab,ti,kw OR 'cerebrovascular disorder':ab,ti,kw OR 'cerebrovascular disorders':ab,ti,kw OR 'cerebrovascular disturbance':ab,ti,kw OR 'cerebrovascular lesion':ab,ti,kw OR 'cerebrovascular pathology':ab,ti,kw OR 'cerebrovascular syndrome':ab,ti,kw OR 'cerebrovasculopathy':ab,ti,kw OR 'brain ischaemic attack':ab,ti,kw OR 'brain ischemic attack':ab,ti,kw OR 'brain transient ischaemic attack':ab,ti,kw OR 'brain transient ischemic attack':ab,ti,kw OR 'cerebral ischaemia, transient':ab,ti,kw OR 'cerebral ischemia, transient':ab,ti,kw OR 'circulatory epilepsy':ab,ti,kw OR 'epilepsy circulatory':ab,ti,kw OR 'ischaemic attack':ab,ti,kw OR 'ischaemic attack, transient':ab,ti,kw OR 'ischaemic cerebral attack':ab,ti,kw OR 'ischemic attack':ab,ti,kw OR 'ischemic attack, transient':ab,ti,kw OR 'ischemic cerebral attack':ab,ti,kw OR 'mini-stroke':ab,ti,kw OR 'transient brain ischaemia':ab,ti,kw OR 'transient brain ischemia':ab,ti,kw OR 'transient cerebral ischaemia':ab,ti,kw OR 'transient cerebral ischemia':ab,ti,kw OR 'transient ischaemic attack':ab,ti,kw OR 'transient ischaemic seizure':ab,ti,kw OR 'transient ischemic seizure':ab,ti,kw OR 'corinthian (device)':ab,ti,kw OR 'e-luminexx':ab,ti,kw OR 'hanaro stent':ab,ti,kw OR 'hanarostent':ab,ti,kw OR 'lifestent':ab,ti,kw OR 'parodi (device)':ab,ti,kw OR 'stenting':ab,ti,kw OR 'stents':ab,ti,kw OR 'aorta coronary artery bypass':ab,ti,kw OR 'aorta coronary bypass':ab,ti,kw OR 'aorta coronary bypass graft':ab,ti,kw OR 'aorta coronary vein bypass':ab,ti,kw OR 'aorta coronary vein bypass graft':ab,ti,kw OR 'aorta coronary vein shunt':ab,ti,kw OR 'aortic coronary artery bypass':ab,ti,kw OR 'aortic coronary bypass':ab,ti,kw OR 'aorticocoronary anastomosis':ab,ti,kw OR 'aorto coronary artery bypass':ab,ti,kw OR 'aorto coronary bypass graft':ab,ti,kw OR 'aorto coronary vein bypass':ab,ti,kw OR 'aortocoronary anastomosis':ab,ti,kw OR 'aortocoronary artery bypass':ab,ti,kw OR 'aortocoronary artery bypass graft':ab,ti,kw OR 'aortocoronary bypass':ab,ti,kw OR 'aortocoronary bypass graft':ab,ti,kw OR 'aortocoronary shunt':ab,ti,kw OR 'aortocoronary vein bypass':ab,ti,kw OR 'aortocoronary vein bypass graft':ab,ti,kw OR 'aortocoronary venous bypass':ab,ti,kw OR 'aortocoronary venous bypass graft':ab,ti,kw OR 'coronary artery bypass':ab,ti,kw OR 'coronary artery bypass grafting':ab,ti,kw OR 'coronary artery graft':ab,ti,kw OR 'coronary bypass':ab,ti,kw OR 'coronary bypass graft':ab,ti,kw OR 'coronary bypass grafting':ab,ti,kw OR 'coronary vein bypass graft':ab,ti,kw OR 'coronary venous bypass graft':ab,ti,kw OR 'ascvd':ab,ti,kw OR 'chd':ab,ti,kw OR 'pad':ab,ti,kw OR 'cva':ab,ti,kw OR 'mi':ab,ti,kw OR 'cad':ab,ti,kw OR 'tia':ab,ti,kw OR 'cabg':ab,ti,kw | 1,241,863 | 1,270,092 |
| #3 | #1 OR #2 | 5,390,616 | 5,590,146 |
| #4 | 'ALN PCSsc':ab,ti,kw OR 'ALN 60212':ab,ti,kw OR 'PCSK9si KJX 839':ab,ti,kw OR 'inclisiran':ab,ti,kw OR 'small interfering RNA':ab,ti,kw OR 'RNAi':ab,ti,kw OR 'siRNA':ab,ti,kw OR 'RNA, Small Interfering':ab,ti,kw | 149,419 | 152,131 |
| #5 | 'crossover procedure':de OR 'double-blind procedure':de OR 'randomized controlled trial':de OR 'single-blind procedure':de OR (random* OR factorial* OR crossover* OR cross NEXT/1 over* OR placebo* OR doubl* NEAR/1 blind* OR singl* NEAR/1 blind* OR assign* OR allocat* OR volunteer*):de,ab,ti | 3,017,340 | 3,091,388 |
| #6 | #3 AND #4 AND #5 | **610** | **678** |

**(c) CENTRAL**

| **Serial** | **Search strategy** | **17 Oct. 2022** | **6 Mar. 2023** |
| --- | --- | --- | --- |
| #1 | MeSH descriptor: [Arterial Occlusive Diseases] this term only | 937 | 1,046 |
| #2 | MeSH descriptor: [Arteriolosclerosis] this term only | 0 | 0 |
| #3 | MeSH descriptor: [Atherosclerosis] this term only | 1,535 | 1,765 |
| #4 | MeSH descriptor: [Coronary Artery Disease] this term only | 7,254 | 8,230 |
| #5 | MeSH descriptor: [Peripheral Arterial Disease] this term only | 1,239 | 1,405 |
| #6 | MeSH descriptor: [Coronary Disease] this term only | 8,122 | 9,149 |
| #7 | MeSH descriptor: [Acute Coronary Syndrome] this term only | 2,328 | 2,638 |
| #8 | MeSH descriptor: [Cardiovascular Diseases] this term only | 9,206 | 11,196 |
| #9 | MeSH descriptor: [Heart Disease Risk Factors] this term only | 214 | 346 |
| #10 | MeSH descriptor: [Angina, Unstable] this term only | 1,081 | 1,255 |
| #11 | MeSH descriptor: [Myocardial Infarction] this term only | 11,161 | 12,963 |
| #12 | MeSH descriptor: [Stroke] this term only | 10,688 | 12,944 |
| #13 | MeSH descriptor: [Cerebrovascular Disorders] this term only | 1,466 | 1,677 |
| #14 | MeSH descriptor: [Peripheral Arterial Disease] this term only | 1,239 | 1,405 |
| #15 | MeSH descriptor: [Ischemic Attack, Transient] this term only | 829 | 956 |
| #16 | MeSH descriptor: [Percutaneous Coronary Intervention] this term only | 2,639 | 3,254 |
| #17 | MeSH descriptor: [Stents] this term only | 3,210 | 3,960 |
| #18 | MeSH descriptor: [Coronary Artery Bypass] this term only | 5,335 | 5,879 |
| #19 | “Diseases, Arterial Occlusive” OR “Disease, Arterial Occlusive” OR “Disease, Arterial Obstructive” OR “Arterial Occlusive Disease” OR “Obstructive Disease, Arterial” OR “Occlusive Diseases, Arterial” OR “Arterial Obstructive Disease” OR “Obstructive Diseases, Arterial” OR “Diseases, Arterial Obstructive” OR “Arterial Obstructive Diseases” OR “Occlusive Disease, Arterial” OR “Arterioloscleroses” OR “Atheroscleroses” OR “Atherogenesis” OR “Artery Diseases, Coronary” OR “Artery Disease, Coronary” OR “Coronary Artery Diseases” OR “Coronary Atherosclerosis” OR “Coronary Arteriosclerosis” OR “Coronary Arterioscleroses” OR “Arterioscleroses, Coronary” OR “Coronary Atheroscleroses” OR “Atherosclerosis, Coronary” OR “Atheroscleroses, Coronary” OR “Arteriosclerosis, Coronary” OR “Left Main Diseases” OR “Left Main Disease” OR “Left Main Coronary Disease” OR “Left Main Coronary Artery Disease” OR “Disease, Peripheral Artery” OR “Peripheral Artery Disease” OR “Diseases, Peripheral Arterial” OR “Disease, Peripheral Arterial” OR “Diseases, Peripheral Artery” OR “Arterial Diseases, Peripheral” OR “Arterial Disease, Peripheral” OR “Peripheral Artery Diseases” OR “Artery Disease, Peripheral” OR “Artery Diseases, Peripheral” OR “Peripheral Arterial Diseases” OR “Coronary Heart Disease” OR “Coronary Heart Diseases” OR “Heart Disease, Coronary” OR “Heart Diseases, Coronary” OR “Disease, Coronary Heart” OR “Coronary Diseases” OR “Diseases, Coronary” OR “Diseases, Coronary Heart” OR “Disease, Coronary” OR “Syndromes, Acute Coronary” OR “Coronary Syndrome, Acute” OR “Syndrome, Acute Coronary” OR “Coronary Syndromes, Acute” OR “Acute Coronary Syndromes” OR “Diseases, Cardiovascular” OR “Disease, Cardiovascular” OR “Cardiovascular Disease” OR “Cardiovascular Risk Score” OR “Risk Score, Cardiovascular” OR “Cardiovascular Risk Scores” OR “Score, Cardiovascular Risk” OR “Cardiovascular Risk, Residual” OR “Risk, Residual Cardiovascular” OR “Residual Cardiovascular Risks” OR “Residual Cardiovascular Risk” OR “Risk Factors for Heart Disease” OR “Risk Factors for Cardiovascular Disease” OR “Factor, Cardiovascular Risk” OR “Risk Factor, Cardiovascular” OR “Cardiovascular Risk Factor” OR “Cardiovascular Risk Factors” OR “Cardiovascular Risk” OR “Risk, Cardiovascular” OR “Cardiovascular Risks” OR “Syndrome, Myocardial Preinfarction” OR “Myocardial Preinfarction Syndromes” OR “Preinfarction Syndrome, Myocardial” OR “Preinfarction Syndromes, Myocardial” OR “Syndromes, Myocardial Preinfarction” OR “Myocardial Preinfarction Syndrome” OR “Unstable Angina Pectoris” OR “Unstable Angina Pectori” OR “Preinfarction Anginas” OR “Angina Pectori, Unstable” OR “Angina, Preinfarction” OR “Anginas, Unstable” OR “Unstable Anginas” OR “Angina Pectoris, Unstable” OR “Angina at Rest” OR “Anginas, Preinfarction” OR “Preinfarction Angina” OR “Unstable Angina” OR “Infarctions, Myocardial” OR “Myocardial Infarctions” OR “Infarction, Myocardial” OR “Infarct, Myocardial” OR “Cardiovascular Strokes” OR “Cardiovascular Stroke” OR “Heart Attack” OR “Heart Attacks” OR “Strokes, Cardiovascular” OR “Stroke, Cardiovascular” OR “Infarcts, Myocardial” OR “Myocardial Infarcts” OR “Myocardial Infarct” OR “Cerebral Strokes” OR “CVAs (Cerebrovascular Accident)” OR “Cerebral Stroke” OR “Strokes, Cerebral” OR “Cerebrovascular Stroke” OR “Strokes, Cerebrovascular” OR “Apoplexy” OR “Stroke, Cerebral” OR “Cerebrovascular Accident” OR “Strokes” OR “Vascular Accidents, Brain” OR “Vascular Accident, Brain” OR “Stroke, Cerebrovascular” OR “Cerebrovascular Strokes” OR “CVA (Cerebrovascular Accident)” OR “Brain Vascular Accident” OR “Apoplexy, Cerebrovascular” OR “Brain Vascular Accidents” OR “Cerebrovascular Apoplexy” OR “Cerebrovascular Accidents” OR “Cerebrovascular Accidents, Acute” OR “Strokes, Acute” OR “Acute Stroke” OR “Acute Cerebrovascular Accidents” OR “Stroke, Acute” OR “Cerebrovascular Accident, Acute” OR “Acute Cerebrovascular Accident” OR “Acute Strokes” OR “Occlusion, Cerebrovascular” OR “Occlusions, Cerebrovascular” OR “Cerebrovascular Occlusion” OR “Cerebrovascular Occlusions” OR “Insufficiency, Cerebrovascular” OR “Cerebrovascular Insufficiency” OR “Insufficiencies, Cerebrovascular” OR “Cerebrovascular Insufficiencies” OR “Vascular Disorders, Intracranial” OR “Cerebrovascular Disorder” OR “Cerebrovascular Disease” OR “Brain Vascular Disorder” OR “Cerebrovascular Diseases” OR “Vascular Disorder, Intracranial” OR “Diseases, Cerebrovascular” OR “Intracranial Vascular Disease” OR “Intracranial Vascular Diseases” OR “Vascular Diseases, Intracranial” OR “Brain Vascular Disorders” OR “Intracranial Vascular Disorder” OR “Vascular Disease, Intracranial” OR “Vascular Disorders, Brain” OR “Intracranial Vascular Disorders” OR “Vascular Disorder, Brain” OR “Disease, Cerebrovascular” OR “Arterial Diseases, Peripheral” OR “Disease, Peripheral Arterial” OR “Peripheral Arterial Diseases” OR “Diseases, Peripheral Arterial” OR “Diseases, Peripheral Artery” OR “Peripheral Artery Disease” OR “Arterial Disease, Peripheral” OR “Artery Diseases, Peripheral” OR “Peripheral Artery Diseases” OR “Artery Disease, Peripheral” OR “Disease, Peripheral Artery” OR “Transient Ischemic Attack, Posterior Circulation” OR “Posterior Circulation Transient Ischemic Attack” OR “Transient Ischemic Attack, Brainstem” OR “Transient Ischemic Attack, Brain Stem” OR “Brain Stem Transient Ischemic Attack” OR “Transient Brainstem Ischemia” OR “Brainstem Transient Ischemic Attack” OR “Ischemia, Transient Brainstem” OR “Brainstem Ischemias, Transient” OR “Brain Stem Ischemia, Transient” OR “Ischemias, Transient Brainstem” OR “Brainstem Ischemia, Transient” OR “Transient Ischemic Attacks, Crescendo” OR “Crescendo Transient Ischemic Attacks” OR “Transient Ischemic Attack, Anterior Circulation” OR “Anterior Circulation Transient Ischemic Attack” OR “TIA (Transient Ischemic Attack)” OR “Attacks, Transient Ischemic” OR “Transient Ischemic Attacks” OR “Transient Ischemic Attack” OR “TIAs (Transient Ischemic Attack)” OR “Ischemic Attacks, Transient” OR “Attack, Transient Ischemic” OR “TIA, Brain” OR “Brain TIA” OR “Ischemias, Transient Cerebral” OR “Transient Cerebral Ischemia” OR “Cerebral Ischemias, Transient” OR “Transient Cerebral Ischemias” OR “Ischemia, Transient Cerebral” OR “Cerebral Ischemia, Transient” OR “Vertebrobasilar Circulation Transient Ischemic Attack” OR “Transient Ischemic Attack, Vertebrobasilar Circulation” OR “Carotid Circulation Transient Ischemic Attack” OR “Transient Ischemic Attack, Carotid Circulation” OR “Percutaneous Coronary Revascularization” OR “Interventions, Percutaneous Coronary” OR “Coronary Revascularizations, Percutaneous” OR “Coronary Revascularization, Percutaneous” OR “Intervention, Percutaneous Coronary” OR “Percutaneous Coronary Interventions” OR “Coronary Intervention, Percutaneous” OR “Coronary Interventions, Percutaneous” OR “Revascularizations, Percutaneous Coronary” OR “Percutaneous Coronary Revascularizations” OR “Revascularization, Percutaneous Coronary” OR “Stent” OR “Artery Bypasses, Coronary” OR “Coronary Artery Bypass Grafting” OR “Aortocoronary Bypasses” OR “Coronary Artery Bypasses” OR “Bypass, Coronary Artery” OR “Bypass Surgery, Coronary Artery” OR “Bypass, Aortocoronary” OR “Bypasses, Coronary Artery” OR “Coronary Artery Bypass Surgery” OR “Bypasses, Aortocoronary” OR “Aortocoronary Bypass” OR “Artery Bypass, Coronary” OR “ASCVD” OR “CHD” OR “PAD” OR “CVA” OR “MI” OR “CAD” OR “TIA” OR “CABG” | 113,178 | 133,841 |
| #20 | # #1 OR #2 OR #3 OR #4 OR #5 OR #6 OR #7 OR #8 OR #9 OR #10 OR #11 OR #12 OR #13 OR #14 OR #15 OR #16 OR #17 OR #18 OR #19 | 136,093 | 155,335 |
| #21 | (“ALN PCSsc” OR “ALN 60212” OR “PCSK9si KJX 839” OR “inclisiran” OR “small interfering RNA” OR “RNAi” OR “siRNA” OR “RNA, Small Interfering”):ti,ab,kw | 429 | 466 |
| #22 | #20 AND #21 | **82** | **97** |

**(d) Web of science**

| **Serial** | **Search strategy** | **17 Oct. 2022** | **6 Mar. 2023** |
| --- | --- | --- | --- |
| #1 | TS=(Arterial Occlusive Diseases OR Arteriolosclerosis OR Atherosclerosis OR Coronary Artery Disease OR Peripheral Arterial Disease OR Coronary Disease OR Acute Coronary Syndrome OR Cardiovascular Diseases OR Heart Disease Risk Factors OR Angina, Unstable OR myocardial infarction OR stroke OR Cerebrovascular Disorders OR peripheral arterial disease OR Ischemic Attack, Transient OR percutaneous coronary intervention OR stents OR Coronary Artery Bypass OR ASCVD OR CHD OR PAD OR CVA OR MI OR CAD OR TIA OR CABG OR Arterial Occlusive Disease* OR Disease*, Arterial Occlusive OR Occlusive Disease*, Arterial OR Arterial Obstructive* Disease OR Disease*, Arterial Obstructive OR Obstructive Disease*, Arterial OR Arterioloscleroses OR Atheroscleroses OR Atherogenesis OR Artery Disease*, Coronary OR Coronary Artery Disease* OR Left Main Disease* OR Left Main Coronary Disease OR Coronary Arteriosclerosis OR Arterioscleroses, Coronary OR Coronary Arterioscleroses OR Atherosclerosis, Coronary OR Atheroscleroses, Coronary OR Coronary Atheroscleroses OR Coronary Atherosclerosis OR Arteriosclerosis, Coronary OR Arterial Disease*, Peripheral OR Disease*, Peripheral Arterial OR Peripheral Artery Disease* OR Artery Disease*, Peripheral OR Disease*, Peripheral Artery OR Peripheral Artery Diseases OR Coronary Diseases OR Disease*, Coronary OR Coronary Heart Disease* OR Disease*, Coronary Heart OR Heart Disease*, Coronary OR Acute Coronary Syndromes OR Coronary Syndrome*, Acute OR Syndrome*, Acute Coronary OR Cardiovascular Disease OR Disease*, Cardiovascular OR Cardiovascular Risk* OR Risk Factor, Cardiovascular OR Risk Factors for Heart Disease OR Risk Factors for Cardiovascular Disease OR Risk Score, Cardiovascular OR Risk, Cardiovascular OR Risk, Residual Cardiovascular OR Anginas, Unstable OR Unstable Angina* OR Angina Pectori*, Unstable OR Unstable Angina Pectori* OR Angina at Rest OR Angina*, Preinfarction OR Preinfarction Angina* OR Myocardial Preinfarction Syndrome* OR Preinfarction Syndrome*, Myocardial OR Syndrome*, Myocardial Preinfarction OR Infarction*, Myocardial OR Cardiovascular Stroke* OR Stroke*, Cardiovascular OR Myocardial Infarct* OR Infarct*, Myocardial OR Heart Attack* OR Strokes OR Cerebrovascular Accident* OR CVA* OR Cerebrovascular Apoplexy OR Apoplexy, Cerebrovascular OR Vascular Accident, Brain OR Brain Vascular Accident* OR Vascular Accidents, Brain OR Cerebrovascular Stroke* OR Stroke*, Cerebrovascular OR Apoplexy OR Cerebral Stroke* OR Stroke*, Cerebral OR Stroke*, Acute OR Acute Stroke* OR Cerebrovascular Accident*, Acute OR Acute Cerebrovascular Accident* OR Cerebrovascular Disorder OR Cerebrovascular Disease* OR Disease*, Cerebrovascular OR Intracranial Vascular Disease* OR Vascular Disease*, Intracranial OR Brain Vascular Disorder* OR Vascular Disorder*, Brain OR Intracranial Vascular Disorder* OR Vascular Disorder*, Intracranial OR Cerebrovascular Insufficienc* OR Insufficienc*, Cerebrovascular OR Cerebrovascular Occlusion* OR Occlusion*, Cerebrovascular OR Arterial Disease*, Peripheral OR Disease*, Peripheral Arterial OR Peripheral Arterial Diseases OR Peripheral Artery Disease* OR Artery Disease*, Peripheral OR Disease*, Peripheral Artery OR TIA* OR Transient Ischemic Attack* OR Attack*, Transient Ischemic OR Ischemic Attacks, Transient OR Cerebral Ischemia*, Transient OR Ischemia*, Transient Cerebral OR Transient Cerebral Ischemia* OR Brain Stem Ischemia, Transient OR Brainstem Ischemia*, Transient OR Ischemia*, Transient Brainstem OR Transient Brainstem Ischemia OR Coronary Intervention*, Percutaneous OR Intervention*, Percutaneous Coronary OR Percutaneous Coronary Intervention* OR Percutaneous Coronary Revascularization* OR Coronary Revascularization*, Percutaneous OR Revascularization*, Percutaneous Coronary OR stent OR Artery Bypass*, Coronary OR Bypass*, Coronary Artery OR Coronary Artery Bypass* OR Aortocoronary Bypass* OR Bypass*, Aortocoronary) | 5,438,902 | 5,560,855 |
| #2 | TS=(ALN PCSsc OR ALN 60212 OR PCSK9si KJX 839 OR inclisiran OR small interfering RNA OR RNAi OR siRNA OR RNA, Small Interfering) | 199,972 | 203,395 |
| #3 | TS=(randomised OR randomized OR randomisation OR randomisation OR placebo* OR (random* AND (allocat* OR assign*)) OR (blind* AND (single OR double OR treble OR triple))) | 1,859,608 | 1,895,690 |
| #4 | #1 AND #2 AND #3 | **475** | **499** |

**(e) Clinicaltrials.gov**

| **Search strategy** | **Filters** | **17 Oct. 2022** | **6 Mar. 2023** |
| --- | --- | --- | --- |
| Other terms: ALN-PCSsc OR ALN-60212 OR PCSK9si KJX-839 OR inclisiran OR small interfering RNA OR RNAi OR siRNA OR RNA, Small Interfering | With Results | **25** | **26** |

**(f) WHO ICTRP**

| **Search strategy** | **Filters** | **17 Oct. 2022** | **6 Mar. 2023** |
| --- | --- | --- | --- |
| ALN-PCSsc OR ALN-60212 OR PCSK9si KJX-839 OR inclisiran OR small interfering RNA OR RNAi OR siRNA OR RNA, Small Interfering | With results only | **9** | **10** |

**Supplementary Table 3.** Conditions of ASCVD or high-risk of ASCVD(Grundy et al., 2019)^a^

|  | **Conditions** |
| --- | --- |
| **ASCVD** | History of acute coronary disease, ischemic stroke, or peripheral artery disease |
| **High-risk of ASCVD** | 1. Age ≥65 years 2. Familial hypercholesterolemia 3. History of prior coronary artery bypass surgery or percutaneous coronary intervention outside of the Major ASCVD events 4. Diabetes mellitus 5. Hypertension 6. Chronic kidney disease (Stage 3 and Stage4) 7. Current smoking 8. History of congestive heart failure 9. Persistently elevated LDL-C (LDL-C ≥100 mg/dL [2.6 mmol/L]) despite maximum tolerated statin therapy and ezetimibe |

*^a^In* ***Supplementary Table 3****, ASCVD atherosclerotic cardiovascular disease, LDL-C low-density lipoprotein cholesterol.*

**Supplementary Table 4.** Definition of stroke or cerebrovascular disease, MACE, and SAE: (a) stroke and cerebrovascular disease; (b) MACE; (c) SAE^a^

**(a)**

| **Stroke and cerebrovascular disease** | **Definition (SMQ/PT)** |
| --- | --- |
|  | Central nervous system vascular conditions (SMQ)   1. Ischaemic central nervous system vascular conditions (SMQ) 2. Haemorrhagic central nervous system vascular conditions (SMQ) 3. Central nervous system vascular disorders, not specified as haemorrhagic or ischaemic (SMQ) |

**(b)**

| **MACE** | **Definition** | **SMQ/PT** |
| --- | --- | --- |
|  | Cardiovascular cause death | / |
|  | Fatal or nonfatal myocardial infarction | Myocardial infarction (SMQ) |
|  | Fatal or nonfatal stroke | Central nervous system vascular disorders (SMQ) |
|  | Cardiac arrest | Cardiac arrest (PT)  Cardio-respiratory arrest (PT) |
|  | Cardiac failure | Cardiac failure (SMQ) |

**(c)**

| **SAE** | **Definition** |
| --- | --- |
|  | Any untoward medical occurrence that at any dose:   1. results in death; 2. is life-threatening; 3. requires inpatient hospitalization or prolongation of existing hospitalization; 4. results in persistent or significant disability/incapacity; 5. is a congenital anomaly/birth defect; 6. Is another medically significant event where medical and scientific judgement should be exercised in deciding whether other situations should be considered serious reactions. |

*^a^In* ***Supplementary Table 4****, MACE major adverse cardiovascular events, SAE serious adverse events, SMQ Standardized medical dictionary for regulatory activities (MedDRA) Queries, PT preferred term.*

**Supplementary Table 5. Definition of stroke, MACE and MI in K et al. study (Ray et al., 2022)** ^a^

| **CV events** | **Definition** |
| --- | --- |
| **MACE** | 1. Cardiac death: Fatal SAEs in cardiac disorders SOC and Fatal SAEs in general disorders SOC (Death, Sudden cardiac death (PT), Cardiac death (PT), Apparent death (PT)) 2. Cardiac arrest: Cardiac arrest (PT) 3. Non-fatal MI: Myocardial infarction (SMQ, broad and narrow) 4. Fatal and non-fatal stroke: Central nervous system haemorrhages (HLT), Cerebrovascular accident (HLT) |
| **Stroke** | Cerebral infarction (PT)  Haemorrhagic stroke (PT)  Ischaemic stroke (PT)  Lacunar stroke (PT)  Cerebrovascular accident (PT) |
| **MI** | Acute myocardial infarction (PT)  Myocardial infarction (PT) |

*^a^In* ***Supplementary Table 5****, CV cardiovascular,PT preferred term, MACE major adverse cardiovascular events, SAE serious adverse event, SOC system organ class, SMQ Standardized medical dictionary for regulatory activities (MedDRA) Queries, HLT high level term, MI: myocardial infarction.*

**(A)**


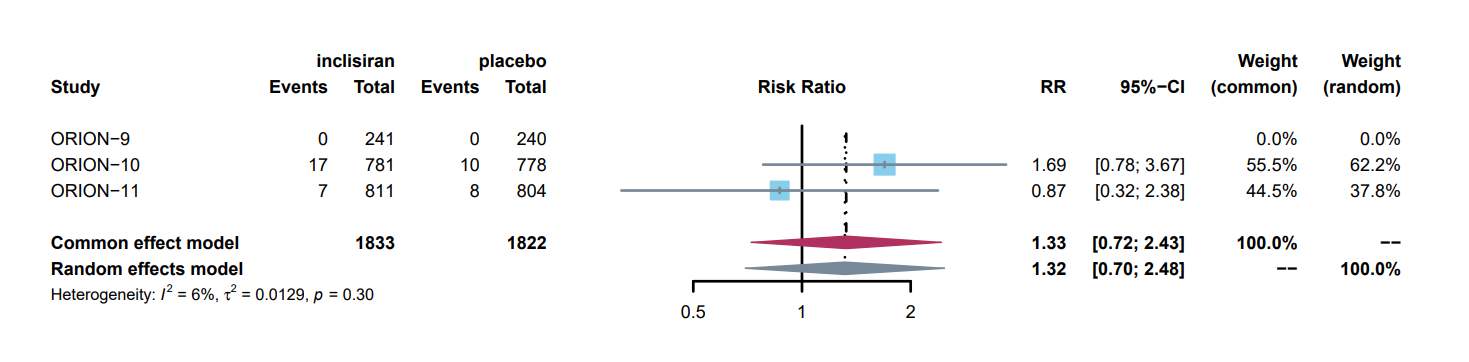


**(B)**


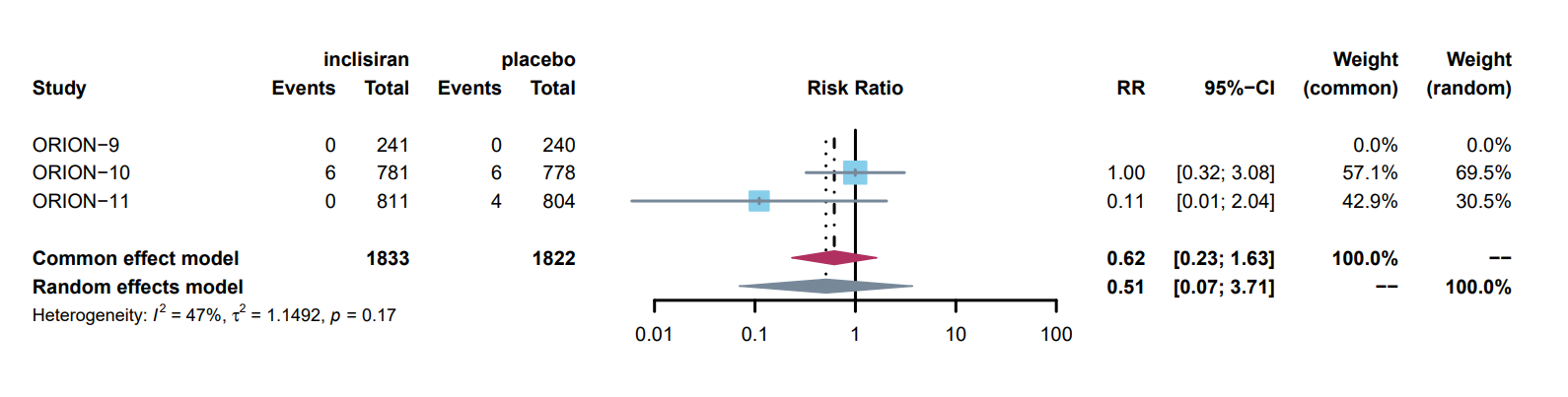


**(C)**


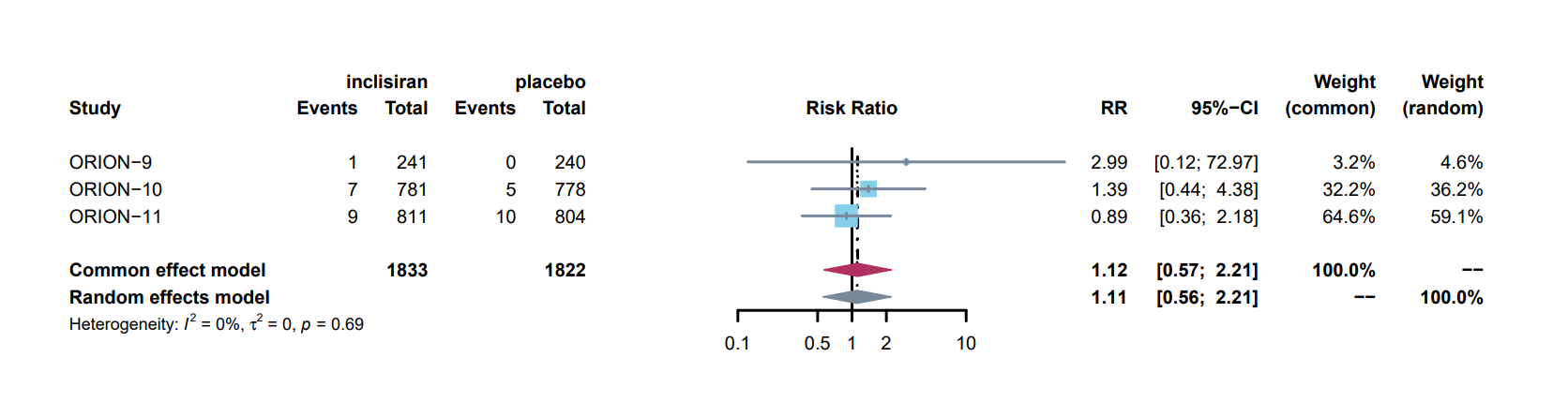


**(D)**


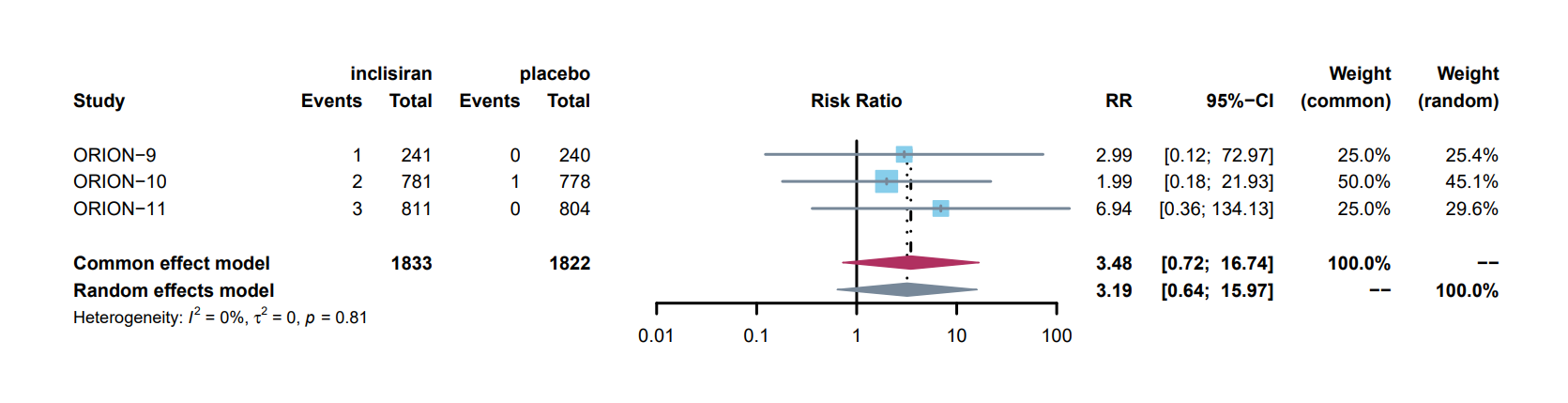


**(E)**

**
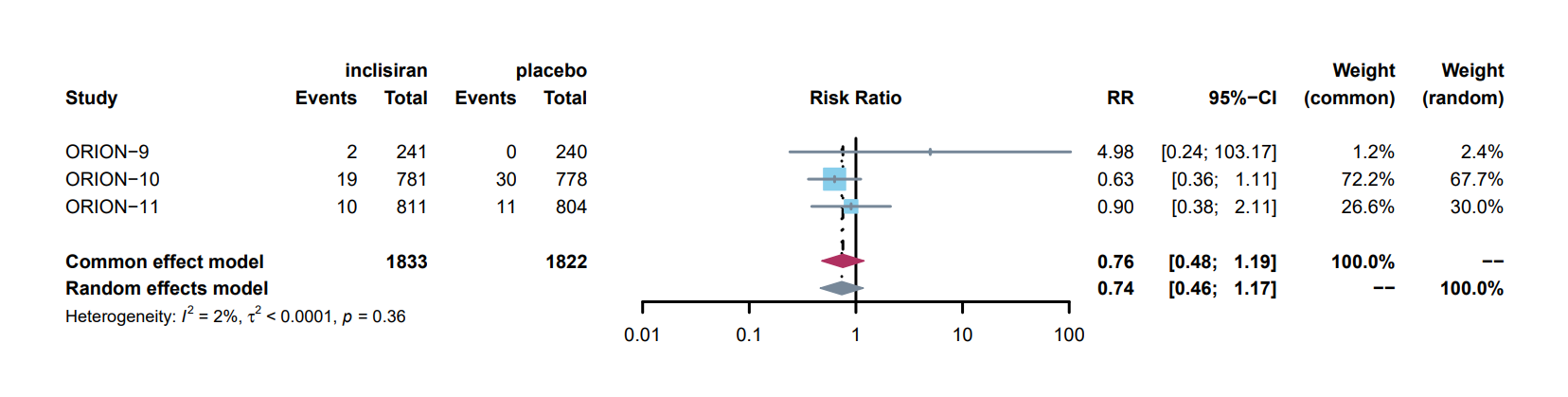
**

**Supplementary Figure 1.** Forest plot of the effect of inclisiran in part component of stroke and MACE, pooled using common-effects meta-analysis: inclisiran intervention versus control group (A) Ischaemic stroke; (B) Haemorrhagic stroke; (C) Cardiovascular cause death; (D) Cardiac arrest; (E) Cardiac failure. Overall, three studies were included in this meta-analysis. The maroon diamond represents the pooled difference using a random effects model for each subgroup and for the total. Heterogeneity for outcomes is represented by I^2^ values (%) with *P* values reported for the χ^2^ test for heterogeneity. The gray diamond represents the result of changing pooled model used to sensitive analysis. *MACE major cardiovascular events, RR risk ratio, CI confidence interval.*

**Reference**

*ICH E2A Clinical safety data management: definitions and standards for expedited reporting - Scientific guideline:* [*https://www.ema.europa.eu/en/ich-e2a-clinical-safety-data-management-definitions-standards-expedited-reporting-scientific*](https://www.ema.europa.eu/en/ich-e2a-clinical-safety-data-management-definitions-standards-expedited-reporting-scientific) [Online]. [Accessed].

Grundy, S.M., Stone, N.J., Bailey, A.L., Beam, C., Birtcher, K.K., Blumenthal, R.S., et al. (2019). 2018 AHA/ACC/AACVPR/AAPA/ABC/ACPM/ADA/AGS/APhA/ASPC/NLA/PCNA Guideline on the Management of Blood Cholesterol: A Report of the American College of Cardiology/American Heart Association Task Force on Clinical Practice Guidelines. *Circulation* 139(25). doi: 10.1161/CIR.0000000000000625.

Page, M.J., McKenzie, J.E., Bossuyt, P.M., Boutron, I., Hoffmann, T.C., Mulrow, C.D., et al. (2021). The PRISMA 2020 statement: an updated guideline for reporting systematic reviews. *Bmj* 372**,** n71. doi: 10.1136/bmj.n71.

Ray, K.K., Raal, F.J., Kallend, D.G., Jaros, M.J., Koenig, W., Leiter, L.A., et al. (2022). Inclisiran and cardiovascular events: a patient-level analysis of phase III trials. *European heart journal*. doi: 10.1093/eurheartj/ehac594.
